# PEACOCK: a machine learning approach to assess the validity of cell type-specific enhancer-gene regulatory relationships

**DOI:** 10.1038/s41540-023-00270-z

**Published:** 2023-04-03

**Authors:** Caitlin Mills, Crystal N. Marconett, Juan Pablo Lewinger, Huaiyu Mi

**Affiliations:** 1 Division of Bioinformatics, Department of Population and Public Health Sciences, Keck School of Medicine, University of Southern California, Los Angeles, CA 90089 USA; 2 Department of Surgery, Keck School of Medicine, University of Southern California, Los Angeles, CA 90089 USA; 3 Department of Biochemistry and Molecular Medicine, Keck School of Medicine USC, Los Angeles, CA USA; 4 Norris Cancer Center, Keck School of Medicine USC, Los Angeles, CA USA; 5 Division of Biostatistics, Department of Population and Public Health Sciences, Keck School of Medicine, University of Southern California, Los Angeles, CA 90089 USA

**Keywords:** Software, Genetic interaction

## Abstract

The vast majority of disease-associated variants identified in genome-wide association studies map to enhancers, powerful regulatory elements which orchestrate the recruitment of transcriptional complexes to their target genes’ promoters to upregulate transcription in a cell type- and timing-dependent manner. These variants have implicated thousands of enhancers in many common genetic diseases, including nearly all cancers. However, the etiology of most of these diseases remains unknown because the regulatory target genes of the vast majority of enhancers are unknown. Thus, identifying the target genes of as many enhancers as possible is crucial for learning how enhancer regulatory activities function and contribute to disease. Based on experimental results curated from scientific publications coupled with machine learning methods, we developed a cell type-specific score predictive of an enhancer targeting a gene. We computed the score genome-wide for every possible cis enhancer-gene pair and validated its predictive ability in four widely used cell lines. Using a pooled final model trained across multiple cell types, all possible gene-enhancer regulatory links in cis (~17 M) were scored and added to the publicly available PEREGRINE database (www.peregrineproj.org). These scores provide a quantitative framework for the enhancer-gene regulatory prediction that can be incorporated into downstream statistical analyses.

## Introduction

Enhancers are short (~50–2000 bp) DNA regulatory elements that activate the expression of target genes in a cell type- and timing-specific manner^1^. Enhancers function independently of orientation and contain transcription factor binding sites, which are used to recruit chromatin remodeling complexes and transcription machinery. The enhancer complex loops over and enters into close physical proximity to the target gene promoter to upregulate transcription^2^. Genes can be targeted by more than one enhancer, and some have been seen to interact with more than ten enhancers^3–5^. A single enhancer may also regulate more than one target gene, perhaps even simultaneously^6^. There is great variability in the distance from which enhancers regulate their target genes. Often an enhancer targets the nearest gene, but enhancers have also been shown to frequently skip the nearest genes to regulate more distal genes, even at distances of over a million base pairs away^7,8^. During cellular differentiation, enhancers play a vital role in cell fate determination^9^. Most cancer cells require the rogue upregulation of oncogenes, which is directed by enhancers or by clusters of enhancers termed super-enhancers, previously described as locus control regions^10–12^. Thousands of disease-associated variants identified by GWAS (genome-wide association studies) have been found to reside in enhancers, implicating them in many deadly diseases^13^.

Although the genomic locations of thousands of enhancers are known, the regulatory target genes of the vast majority of enhancers are unknown^14^. Determining which genes are the regulatory targets of specific enhancers—especially those already associated with the disease—may be the key to learning how enhancer regulatory activities function within and contribute to many deadly diseases.

Since it is widely believed that enhancers come into close physical proximity with the promoters of their target genes in order to regulate their transcription, some of the most common experimental methods by which enhancer-gene regulatory associations are identified are via proximity ligation assays (e.g., 3 C, 4 C, 5 C, Hi-C, promoter-capture HiC, ChIA-PET, Hi-ChIP, etc.) and advanced microscopy techniques which seek to identify long-range physical interactions between enhancers and promoters to establish a regulatory relationship^15^. Genome and epigenome editing techniques, as well as experimental engineering of enhancer-promoter interactions (e.g., CRISPR-based techniques), provide excellent avenues for elucidating specific enhancer-gene regulatory relationships in specific cell types^15^. However, these experimental approaches do not scale genome-wide; thus, the vast majority of the targets remain unknown. Due to the incredibly cell type-specific nature of enhancer-gene regulatory relationships, the genome-wide characterization of enhancer-gene regulatory networks in a large range of cell types is a significant need in the advancement of disease research.

Many computational efforts to predict large quantities of enhancer-gene regulatory links based on publicly available experimental data^16–21^ have resulted in numerous databases with predictions of enhancer-gene activity. However, these approaches often lack a mechanism to validate the accuracy of their predictions. There is not enough cell type-matched data provided in these databases on which target genes are regulated by specific enhancers to be able to statistically validate any score (when one is provided) of these predicted enhancer-gene regulatory links, which adds a great degree of uncertainty to such datasets. Although there are instances of prediction databases capturing known examples of enhancer-gene regulatory relationships, the proportion of spurious enhancer-gene links included along with the legitimate ones in these sets of predictions is usually unknown.

Although much is known about which characteristics are important to identifying enhancer-gene regulatory links, it is not known how to weigh these different characteristics simultaneously. Thus, manually programing a set of rules to identify active gene-enhancer pairs for all possible configurations of inputs is not feasible. Instead, supervised machine learning allows us to train an algorithm by showing it examples of the desired input-output behavior. Specifically, the task of predicting whether an enhancer targets a particular gene, can be cast as a binary classification problem. In a binary classification setting, an algorithm is trained to classify new possible enhancer-gene links as a member of one of the two classes (positive or negative, where positive indicates that the enhancer-gene link is a true active regulatory relationship and negative indicates that the enhancer-gene pair has no active regulatory relationship) based on characteristics of the enhancer (e.g., H3K27ac marks), characteristics of the gene (e.g., H3K4me3 marks), and characteristics of the pair (e.g., statistically significant eQTL for the gene mapped within the enhancer). Probabilistic classifiers output a quantitative score that can be thresholded to produce a predicted class membership: above the threshold to one class and below to the other.

**Fig. 1:**
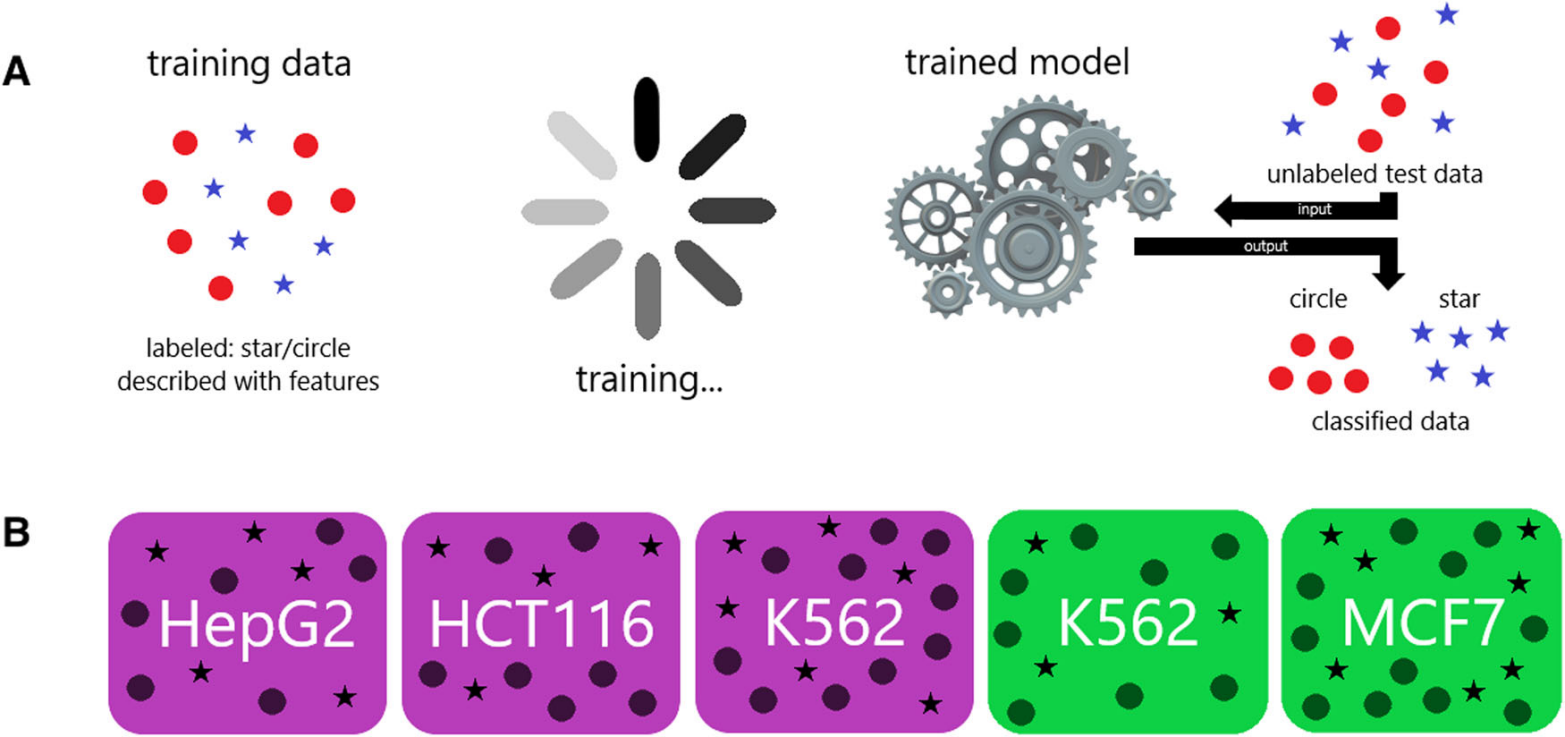
Schematic of training and testing the models. The pipeline illustrated here is for the classification of enhancer-gene pairs into active vs. inactive. The training data consists of examples of active enhancer-gene pairs (links) and inactive enhancer-gene pairs represented by stars and circles. Panel **A** shows the training data as labeled (i.e., the model is able to see which enhancer-gene pair belongs to the active and inactive class) and described features believed to be predictive of activity status. In the training phase, a machine learning algorithm learns which patterns in the feature data best differentiates the two classes and builds a predictive model. The trained model is then able to take new, unlabeled data as input and output a classification score based on what it learned in the training phase. Test data not used in the training phase is used to unbiasedly measure the prediction performance of the trained model, where the class of each observation is known to the user but unknown to the classifier. In this way, it is possible to determine how well the model classifies new data. **B** In this analysis, there were three training datasets (purple) from three different cell lines. There are also two test datasets which were never used for model training (green), which come from two cell types. If a training dataset is not used for training the model, it may also serve as a testing dataset for a model using a different dataset to train on. For example, a model which uses HepG2 training data to train on may use any of the other datasets for testing. The same observations are never used for both training and testing a model.

We developed a simple yet effective approach (PEACOCK: **P**redicted **E**nhancer **A**ctivity in **C**is **O**riginating from **C**ell-specific **K**nowledge) to predict gene-enhancer regulatory links using machine learning classification algorithms. Peer-reviewed scientific articles in four cancer cell lines (HepG2 (liver cancer), HCT116 (colorectal cancer), K562 (leukemia), and MCF7 (breast cancer)) were scoured to amass 159 experimentally validated enhancer-gene links to generate cell type-specific positive enhancer-gene regulatory examples. Publicly available DNA accessibility data in the same cell lines were leveraged to generate a large collection of negative examples. Machine learning models were trained separately on the training data generated for each cell type and evaluated on their ability to identify true (or active) enhancer-gene regulatory links on separate test data (Fig. 1).

For each enhancer-gene pair, characteristics (called “features” in machine learning) that are widely believed to be hallmarks of enhancer activation of target genes were used to describe the potential regulatory link of the pair. Well-chosen features enable the machine learning algorithms to identify meaningful patterns that distinguish between the positive enhancer-gene links representing a true regulatory relationship and the negative enhancer-gene links representing an enhancer-gene pair with no regulatory relationship in a particular cellular environment. ChIP-seq data from ENCODE targeting epigenetic marks associated with active enhancers (H3K27ac, H3K4me1, and binding of histone acetyltransferase P300) and actively regulated promoters (H3K4me3 and H3K27ac) were included. A measure of significance (*p* value) and a measure of effect (regression coefficient) for eQTL from GTEx^22^ which mapped to enhancers, were also included as potentially predictive features. Additionally, two binary features recorded whether the gene was located nearest to the enhancer (yes vs. no) and whether the enhancer was located in one of the gene’s introns (yes vs. no) (Table 3).

Enhancer-gene pairs with these features were used as training examples to allow the classification algorithms to learn from the data. Each algorithm was evaluated on test data that was not used in the training phase. Based on the prediction performance of each algorithm on previously unseen test datasets from multiple cell lines, a final model was selected for use in predicting a score for new enhancer-gene pairs, where a higher score represents a greater chance of the enhancer-gene pair having an active regulatory relationship in a given cell type.

The final models perform well both within and across cell types, even from highly unrelated tissue types. Our findings suggest that although the behavior and state of individual enhancer-gene pairs are highly specific to the particular cell type at hand, the characteristics of active enhancer regulation of target genes remain consistent across cell types. It is, therefore, possible to pool observations from multiple cell types to make a larger training dataset as long as within-observation cell type specificity remains consistent. Using a pooled final model trained across multiple cell types, all possible gene-enhancer regulatory links in cis (~17 M) were scored (Supplementary Figs. 1–7) and added to the previously described PEREGRINE^23^ database (www.peregrineproj.org).

Whether on the scale of a single enhancer-gene pair or the entire genome, these scores provide an accurate and consistent measure with which investigators can evaluate possible target genes of specific enhancers in a particular cellular environment. Applications range from prioritization of enhancer-gene pairs for experimental validation at the bench, to incorporation into downstream statistical analyses of disease-associated variants.

## Results

### Training and testing datasets

Enhancer-gene regulatory links curated from the scientific literature with a defined set of criteria (Table 1, Supplementary Table 1 of Supplementary Materials) made up the positive class of the training or test dataset for each cell line (HepG2: *n* = 23, HCT116: *n* = 43, K562_1: *n* = 60, K562_2: *n* = 6, MCF7: *n* = 27). The negative class was annotated using DNA accessibility data, comprising 15,491,123 enhancer-gene pairs in HepG2 data, 16,304,125 enhancer-gene pairs in HCT116 data, 13,205,263 enhancer-gene pairs in K562 data, and 16,215,821 enhancer-gene pairs in MCF7 data. A subset of observations from the negative class were selected for use in analysis based on their genomic proximity to the positive observations (HepG2: *n* = 360, HCT116: *n* = 420, K562_1: *n* = 400, K562_2: *n* = 1303, and MCF7: n = 300).

**Table 1:** Description of the positive set criteria.

Criterion 1	CRISPR deletion or mutation of the enhancer results in statistically significantly altered expression of the target gene.
Criterion 2	Transcription factors that help upregulate the expression of the target gene were shown to interact with the enhancer. Mutating the transcription factor binding sites within the enhancer resulted in statistically significantly altered target gene expression.
Criterion 3	SNPs located in an enhancer alter target gene transcription statistically significantly in an allele-specific manner. The enhancer was shown to interact physically with the target gene’s promoter. The SNPs were observed in GWAS to be associated with differential expression of the target gene.
Criterion 4	A transcription factor known to be important to the expression of the target gene is shown to bind to the enhancer. Knockdown experiments of the transcription factor are shown to statistically significantly decrease the target gene’s expression. SNPs located in an enhancer alter target gene transcription statistically significantly in an allele-specific manner.
Criterion 5	The enhancer was shown to confer an inducible expression of the target gene. Binding motifs were found in the enhancer for a transcription factor that was shown to greatly alter inducible expression in the presence of an expression vector for the transcription factor. Deletions within the enhancer of these binding motifs altered gene expression.

These are the different criteria that enable an enhancer-gene link from the literature to be accepted into the positive class in the training dataset and used for analysis. NOTE: Although Criterion 1 could lead to the inclusion of indirect effects (e.g., CRISPR deletion of an enhancer effecting gene A which then effects gene B), we believe that these kinds of indirect effects may still be useful in elucidating an enhancer’s regulatory involvement in a biological process, even if it is of a more upstream nature than can be captured by these predictions. Regardless, the user should be made aware of this limitation.

### Features

Characteristics that are classically associated with active enhancer regulation of target genes (e.g., acetylation and histone methylation marks) were gathered to be used as features (variables) in the predictive models. Features we considered for an enhancer-gene pair included characteristics that are classic hallmarks of the active state of the enhancer, the active state of the gene, as well as some features that are thought to indicate regulatory activity between the enhancer and the gene. There are nine main features in the cell type-specific datasets of cis enhancer-gene pairs (Table 2). To capture the enhancer-gene pair as a unit, rather than just the enhancer or just the gene, we also defined new features consisting of interaction terms between the enhancer-specific features and the promoter-specific features (Table 3). This resulted in up to 17 total features for each dataset of cell type-specific enhancer-gene pairs. All 17,354,145 enhancer-gene pairs made up of an enhancer-gene pair located <1 Mb apart were then attributed values for all available features.

**Table 2:** Description of the main effect features.

Feature (enhancer, gene, or both)	Description	Value
Feature 1: H3K27ac (Enhancer)	A histone acetylation mark commonly associated with active enhancers	0/1 (binary for high value or not the high value of the peak of ChIP-seq peak)*
Feature 2: H3K4me1 (Enhancer)	A histone methylation mark commonly associated with active and poised enhancers	Positive continuous value (score of the ChIP-seq peak)
Feature 3: H3K4me3 (Promoter)	A histone methylation mark commonly associated with the promoters of genes being actively enhanced	0/1 (binary for high value or not the high value of the peak of ChIP-seq peak)*
Feature 4: P300 binding (Enhancer)	Binding of P300, a histone acetyltransferase well established as a marker of active enhancers^44^, to the enhancer	Positive continuous value (score of the ChIP-seq peak)
Feature 5: eQTL—combined *Z*-score	eQTL *p* values transformed (see Methods) into a combined *Z*-score for each enhancer with eQTL(s) pointing to the same gene (a measure of statistical significance)	Positive continuous value
Feature 6: nearest gene	Is the gene in this link the enhancer’s nearest gene?	0/1 (binary, 0 = no, 1 = yes)
Feature 7: intronic	Is the enhancer located in an intron of the target gene?	0 = enhancer not located in an intron of the target gene 1 = enhancer located in the intron of the target gene
Feature 8: average of the absolute values of eQTL coefficients of eQTL located within the enhancer pointing to the same gene	If multiple eQTL for the same gene are located in the same enhancer, what is the average value of the absolute values of the coefficients (a measure of effect) of these eQTL? (see Methods)	Positive continuous value
Feature 9: H3K27ac (Promoter)	A histone acetylation mark commonly associated with promoters of genes being actively enhanced	Positive continuous value (score of the ChIP-seq peak)

*The threshold for classifying a value as “high” is described in the Methods for ENCODE’s Registry of candidate cis-regulatory elements (cCREs) where this data were collected from.

All of the features listed are from ENCODE datasets from HepG2, HCT116, K562, and MCF7 cells, with the exception of eQTL data which was only available from GTEx at the tissue level (liver, colon, whole blood, and breast, were used respectively) and P300 data which was unavailable in HCT116. For any features involving the overlap of a ChIP-seq peak signal (H3K27ac, H3K4me1, H3K4me3, and P300 binding), overlap between the two features (enhancer/gene and peak) was required at a minimum threshold of 50% overlap for either region.

**Table 3:** Interaction terms between active enhancer and promoter marks.

	Promoter marks		
**Enhancer marks**		**H3K27ac**	**H3K4me3**
	**H3K27ac**	H3K27ac_enhancer_ * H3K27ac_promoter_	H3K27ac_enhancer_ * H3K4me3_promoter_
	**H3K4me1**	H3K4me1_enhancer_ * H3K27ac_promoter_	H3K4me1_enhancer_ * H3K4me3_promoter_
	**H3K27ac, H3K4me1**	H3K27ac_enhancer_ * H3K4me1_enhancer_ * H3K27ac_promoter_	H3K27ac_enhancer_ * H3K4me1_enhancer_ * H3K4me3_promoter_

The columns are headed by epigenetic histone marks commonly associated with active genes, while the rows are headed by epigenetic histone marks commonly associated with active enhancers. The box at the intersection of a row and a column is filled in with the interaction term, which is a product of the active promoter mark described at the top of that column and the active enhancer mark described at the beginning of that row.

### Model generation and evaluation

For each cell type-specific training set in K562_1, HepG2, and HCT116, a wide range of machine learning models were trained, including random forests, flexible discriminant analysis, linear discriminant analysis, gradient boosting machines, ridge regression, k-nearest neighbor, and support vector machine models with Gaussian radial, polynomial, linear, hyperbolic tangent, Laplace radial, Bessel, and ANOVA radial basis kernels^24^. Each cell type-specific trained model was evaluated in terms of the Area Under the Precision-Recall Curve (AUPRC) and the Area Under the Receiver Operating Curve (AUC) values they achieved on MCF7 and K562_2 previously unseen test sets. Models were also evaluated on their prediction performance using any of the datasets from HepG2, HCT116, or K562_1 that did not serve as training data for that model (i.e., a model trained on HepG2 data could be tested on the HCT116 dataset, the K562_1 dataset, the K562_2 dataset, or the MCF7 dataset.).

**Fig. 2:**
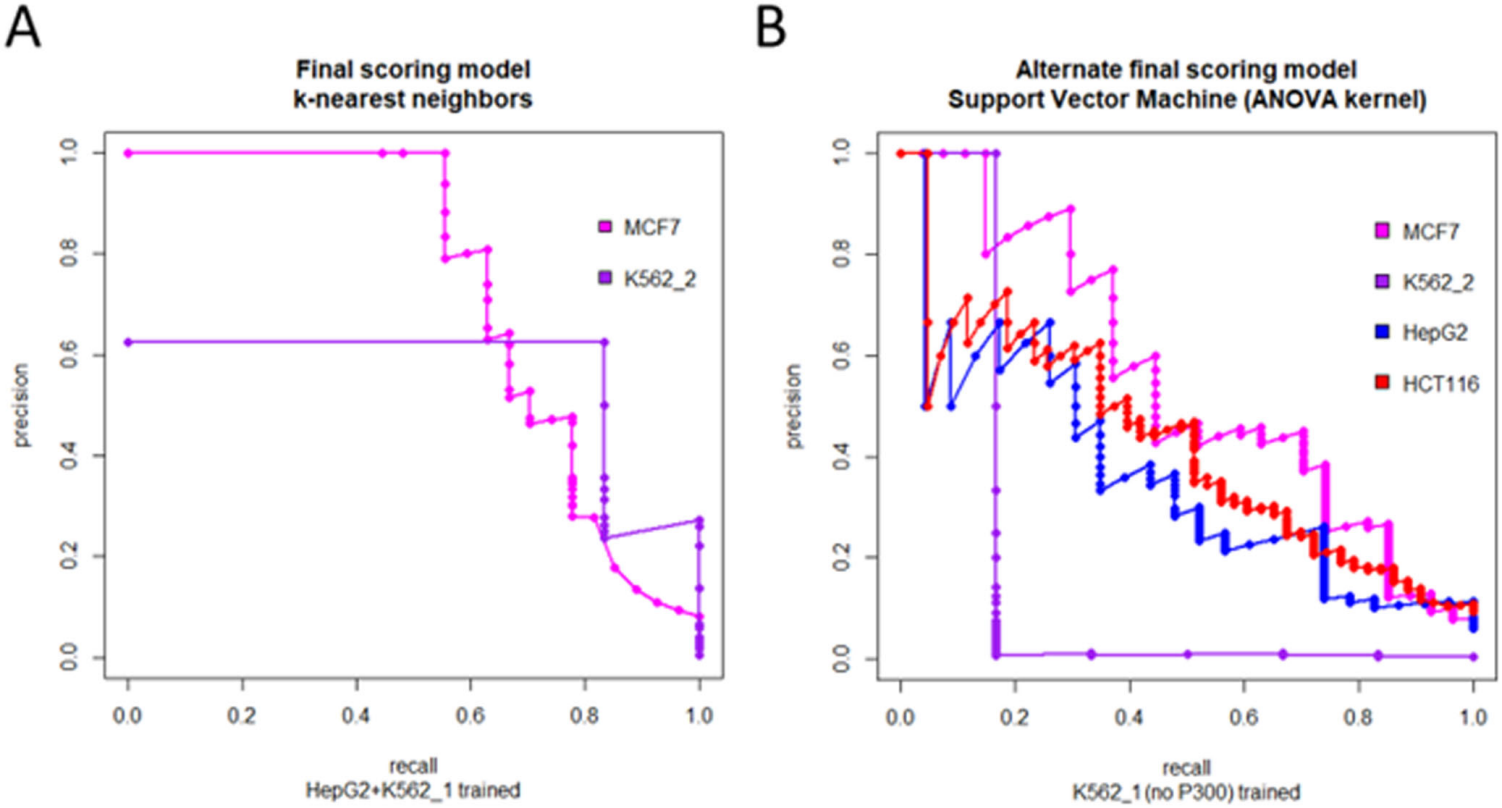
Precision-recall curves of the best-performing models on test datasets. The precision-recall curve (PRC) is given for each model and all test sets available for that model. **A** PRCs for the best final model (k-nearest neighbors) which was trained on the HepG2 + K562_1 joint dataset, evaluated on the MCF7 (AUPRC: 0.73) and K562_2 (AUPRC: 0.56) test datasets. **B** PRCs for the best alternate final model (support vector machine with an ANOVA radial basis kernel), which was trained on the K562_1 set and tested on the MCF7 (AUPRC: 0.55), K562_2 (AUPRC: 0.17), HepG2 (AUPRC: 0.36), and HCT116 (AUPRC: 0.42) test datasets. This model was based on training data that did not include the P300 feature and is only suitable for scoring enhancer-gene links for which P300 data were unavailable.

Remarkably, models that were trained in one cell type were found to perform quite well on test data from a completely unrelated cell type (Fig. 2, Supplementary Tables 3–7, and Supplementary Materials), suggesting that although the specific activities of individual enhancers are very cell type-specific due to the sometimes dramatic differences in the cellular environment from one cell type to another (Supplementary Figs. 11–16), the overall patterns of the relevant features of enhancer regulatory activity remain consistent across cell types. Thus, we explored pooling enhancer-gene pair data from more than one cell type to develop a jointly trained model that can gain from the added robustness of a larger training dataset (Supplementary Tables 8–12 and Supplementary Materials).

### Prediction performance

There are many metrics for assessing the prediction performance of different models, and no universally best one. Because in this application, the relative size of the positive and negative classes (active vs. inactive gene-enhancer pairs) is imbalanced, it is important to consider which class a misclassification error occurs in. In our setting, it is far preferable to have a false negative (an active gene-enhancer pair declared inactive) than a false positive (an inactive gene-enhancer pair declared active), as false positives are much more likely to result in significant cost to further characterize these predictions, and negative downstream impact of pursuing a wrong lead. Because true enhancer-gene regulatory links are rare compared to enhancer-gene pairs with no regulatory relationship (In our K562_2 dataset of 1309 observations based on two genomic regions totaling ~2 Mb altogether, the positive class made up less than 0.5% of total enhancer-gene pairs.), it is possible to have relatively few misclassification errors as long as the negative class has a lower error rate to counterbalance the higher error rate in the comparatively small positive class. Consequently, we evaluated model performance based on AUPRC, which is suitable for imbalanced settings as it ensures good performance on the (smaller) positive class. Precision (sensitivity) is the proportion of predicted positive enhancer-gene links that are actually active. Recall (positive predictive value) is the proportion of active enhancer-gene pairs were correctly predicted as true. As a supplementary performance metric, we also present the AUC. The AUPRC and AUC were measured for every available test dataset and used to select a final model suitable for predicting new enhancer-gene links.

### Model selection

Since models trained in one cell type were shown to be capable of excellent prediction performance in test data from other cell types (with AUPRCs as high as 0.77 and AUCs frequently over 0.90), the final model chosen to be used for predicting new enhancer-gene links was chosen not only based on how well it predicted (as measured by AUPRC and AUC), but how consistently it predicted well across all available test sets (Fig. 2). Any model that performed poorly in at least one test set was not considered suitable for selection as the final model. The support vector machine models with Laplace kernels were the best-performing models when trained in HepG2 and K562_1, and these models performed consistently well across all cell types (Supplementary Tables 4, 7 of Supplementary Materials). Among the jointly trained models, the models trained in the HepG2 + K562_1 joint training dataset performed the best across all test sets in a consistent manner (Supplementary Table 9 of Supplemental Materials). The best performing HepG2 + K562_1 trained model (using a k-nearest neighbors classifier) was then submitted to feature selection by dropping each feature one by one and evaluating the AUPRC in the smaller model. The model performed slightly better without Feature 11 (Supplementary Table 16). Further dropping of features was not found to increase prediction performance (Supplementary Table 17). Consequently, this model was selected as the final model to score enhancer-gene links (Fig. 2A).

Many commonly used cell lines contain nearly the full set of features needed for the final model, but they are missing ChIP-seq data targeting histone acetyltransferase P300, rendering the HepG2 + K562_1 final model unusable. This was true for the HCT116 dataset, so an alternate final model was developed in an analogous manner for cell types missing P300 data to score cell lines for which no P300 ChIP-seq data is available. K562_1 and HepG2 training sets excluding the P300 feature, were generated for this process. Although these models generally performed worse than the models which were able to utilize the P300 feature, it is still important to be able to score enhancer-gene links without P300 data. The support vector machine model with an ANOVA radial basis kernel trained using the K562_1 dataset performed the best across all test sets and was selected as the alternate final model for use in scoring enhancer-gene links in cell lines for which no P300 data is available (Fig. 2B).

In order to make the cell type-specific scores more interpretable across cell types, the cell type-specific *Z*-score (see Methods) is provided for each cis enhancer-gene pair in addition to the raw score (in the [0,1] interval), which should only be used to compare enhancer-gene pairs scored in the same cell type. Although a higher cell type-specific score represents a higher chance of the enhancer-gene pair representing an active regulatory relationship in that cell type, this score should not be interpreted as a probability because the positive (active) and negative (inactive) classes in the training data were not sampled proportionally to their true proportion among all cis gene-enhancer pairs (which is not known). To supplement the raw score, the percentile of each score (*F* score) is also presented. Thus, an *F*(score) = 0.99 is higher than 99% of all scores in the cell type). Cell type-specific scores, *Z*-scores, and *F*(scores) for all possible cis enhancer-gene regulatory links are available at the PEREGRINE website for bulk download (www.peregrineproj.org) in four cell types, which is continuously updated as more publicly available data on diverse cell types using the indicated marks becomes available. The cell type-specific scores, *Z*-scores, and *F*(scores) for PEREGRINE enhancer-gene links are also available in the new Enhancer module of the PANTHER^25^ website (www.pantherdb.org), where the user may easily access the supporting evidence for each PEREGRINE enhancer-gene link, as well as query by target gene and enhancer location.

### PEREGRINE enhancer-gene links have higher cell type-specific scores than other possible enhancer-gene links in cis

**Fig. 3:**
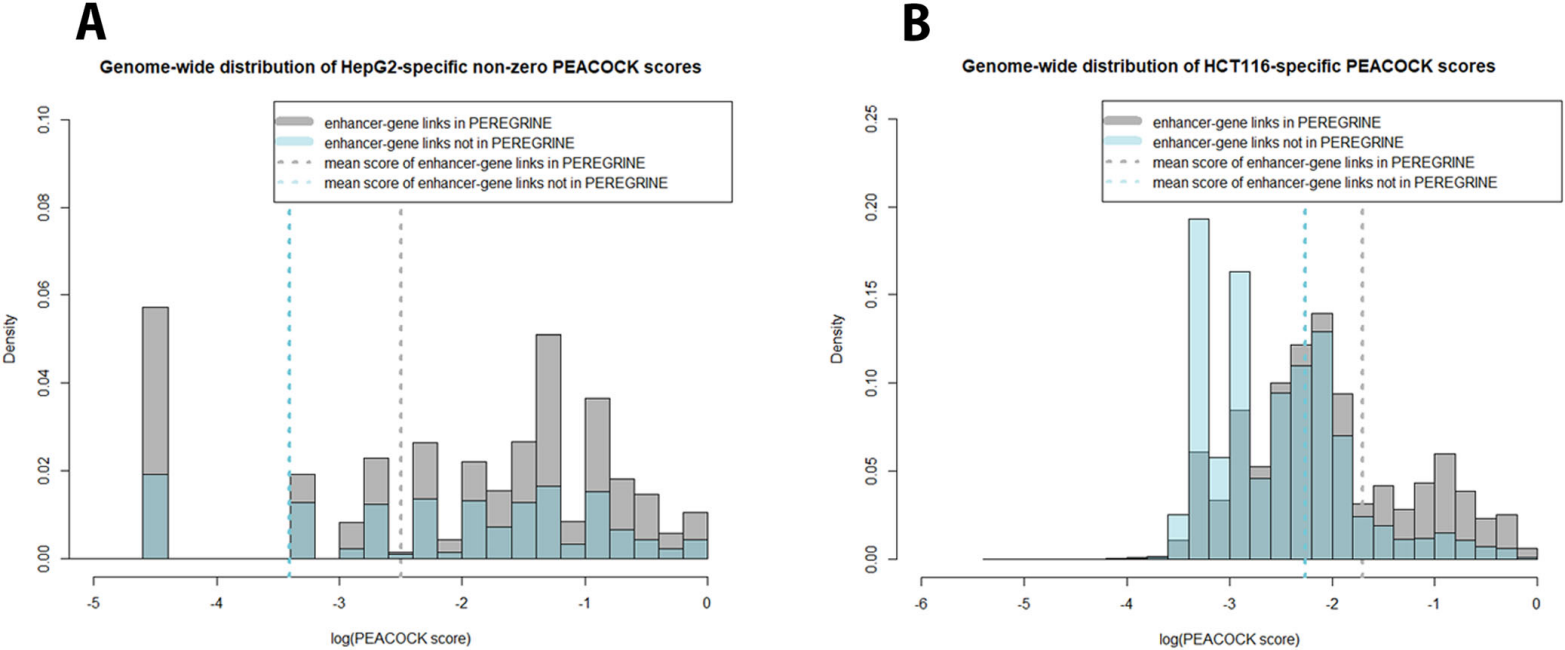
Predicted cell type-specific scores for all possible enhancer-gene links in cis. All possible cis enhancer-gene links (enhancer and gene pairs located <1 Mb apart) are separated by those predicted in PEREGRINE (magenta), and those that were not predicted in PEREGRINE (purple) for HepG2 (**A**) and HCT116 (**B**) cell-specific data. The histograms of each population are overlaid. The mean of each population’s cell type-specific scores is plotted as a vertical bar along the histogram. **A** shows the distribution of the non-zero PEACOCK scores, which makes up about 84% of pairs genome-wide. **B** shows the overall distribution, as no predictions were scored as zero. Distributions for scores in K562 and MCF7 were nearly identical to that of HepG2 and so are not shown here (Supplementary Figs. 7–12 of Supplementary materials). Two-sample KS tests show that for all cell types, the distributions of PEREGRINE cis enhancer-gene links are statistically significantly different than the distributions of the non-PEREGRINE cis enhancer-gene pairs (*p* < 2.2e-16), and in all cell lines the score means are higher for PEREGRINE enhancer-gene links.

The enhancer-gene link database PEREGRINE, a genome-wide prediction of enhancer-to-gene relationships supported by experimental evidence, was recently published^23^. These predicted links were based on publicly available experimental data from ChIA-PET, eQTL, and Hi-C assays across 78 cell and tissue types. Unfortunately, few of the experiments were available in the same cell or tissue types as the other assays, making it difficult to amass cell type-matched evidence for cell type-specific predicted enhancer-gene regulatory links. Although *p* values were reported for experimental evidence whenever available from their original sources, PEREGRINE lacked a statistically validated cell type-specific score for its predicted enhancer-gene regulatory links, a common shortcoming among enhancer-gene regulatory prediction databases. Using data for all cis enhancer-gene pairs, cell type-specific scores generated from the final model (if all features were available) or the alternate final model (if P300 data was unavailable) were attributed to all 17 M possible enhancer-gene link pairs. The distribution of the scores for enhancer-gene links found in PEREGRINE was compared to the distribution of the scores for all other possible cis enhancer-gene links not reported in PEREGRINE (Fig. 3). The scores among PEREGRINE links were markedly higher than the scores among the remaining cis enhancer-gene pairs that were not reported in PEREGRINE in all cell types. The probability density plots for non-PEREGRINE pairs have much higher peaks near zero than the distribution for PEREGRINE link scores (Fig. 3A–D). Two-sample Kolmogorov–Smirnov tests show that for all cell types, the scoring distributions of PEREGRINE cis enhancer-gene links are statistically significantly different than the scoring distributions of the non-PEREGRINE cis enhancer-gene pairs (*p* < 2.2e-16), and in all cell types the score means are higher for PEREGRINE enhancer-gene links. These findings suggest that enhancer-gene links predicted by PEREGRINE have a much higher chance of representing real enhancer-gene regulatory relationships than enhancers and genes located less than 1 Mb apart but not predicted by PEREGRINE.

### Comparison with other methods

PEACOCK predictions scored using the final model were compared with other available approaches, including a random classifier, distance-only model, and previously published predictions from the Activity-By-Contact (ABC) model^26^, GeneHancer^17^, and TargetFinder^14^ (Table 4).

**Table 4:** Prediction performance for other approaches.

Method	HepG2	K562_1	K562_2	HCT116	MCF7
Random classifier	0.06	0.13	0.005	0.09	0.08
Distance-only	0.50	0.55	0.03	0.43	0.38
ABC PEACOCK			0.47 0.68		
GeneHancer PEACOCK			0.04 0.45		
TargetFinder PEACOCK			0.04 0.33		

Each method is listed on the left, and the AUPRC is reported for each method. Columns denote the test datasets. Merged cells indicate combined test datasets. ABC and TargetFinder did not report predictions for any cell type available in PEACOCK except for K562. For previously published methods, the AUPRC was measured on the subset of data with predictions available in both PEACOCK and the comparator. Therefore, PEACOCK performance in K562 varies depending on which method is being compared. GeneHancer predictions are not cell-specific, so the predictions were applied to all cell types used for testing and merged into one dataset for measuring AUPRC. The merging of the three test sets was due to only a few predictions per dataset being available in both PEACOCK and GeneHancer. For distance-only models, the AUPRC of the best-performing model was reported. For the models tested in HepG2 and K562_2, the training sets for those best-performing models were HCT116. For the models tested in HCT116, K562_1, and MCF7, the training sets for those best-performing models were HepG2. Greater detail is reported in the Supplementary Materials and Methods sections.

The AUPRC for the random classifier model represents the baseline AUPRC in each dataset or the proportion of positive examples in the dataset. Any AUPRC greater than this baseline is considered to have some predictive value. PEACOCK’s final model generates AUPRCs much higher than the random classifier and distance-only models on all available test datasets (Fig. 3A). PEACOCK also outperforms GeneHancer (AUPRC = 0.04), TargetFinder (AUPRC = 0.04), and ABC (AUPRC = 0.47) by a wide margin on datasets made up of only predictions available in both PEACOCK and the comparison method being evaluated (PEACOCK AUPRC = 0.45, 0.33, and 0.68 respectively).

### Systems biology implications

In an effort to contextualize the regulatory landscape of the cells scored in PEACOCK, some basic global analyses were conducted. Although the user can select any cutoff value that they want, we used enhancer-gene links with an *F*(score) of no less than 0.95 (corresponding to the top 5% highest scoring enhancer-gene links in each cell line) to generate these statistics. The proportion of enhancers linked to more than one gene as well as the enhancers linked to the most genes in each cell line are reported. Similarly, the proportion of genes predicted to be regulated by more than one enhancer as well as the gene predicted to be regulated by the most enhancers, are reported in Table 5.

**Table 5:** Genome-wide enhancer-gene regulatory summary.

	HepG2	HCT116	K562	MCF7
Enhancers linked to >1 gene	28.5%	7.93%	27.3%	30.1%
Number of genes linked to >1 enhancer	98.7%	98.2%	98.9%	98.6%
Gene linked to the most enhancers	GSDMC (608 enhancers)	LPP (731 enhancers)	KCNMB2 (568 enhancers)	PTPRC (535 enhancers)
Enhancer linked to the most genes	EH37E0433320 (93 genes)	53166, 53167, EH37E0835389, EH37E0835393 (77 genes)	EH37E0433708, 24552 (99 genes)	EH37E0433775 (102 genes)

Enhancer-gene pairs with *F*(score) ≥0.95 (corresponding to the top 5% of all predictions) were used to calculate these statistics. In the event of a tie, all winners are listed.

## Discussion

### Using the cell type-specific enhancer-gene link scores

Cell type-specific enhancer-gene link scores generated via PEACOCK can be accessed in various ways. On the PANTHER website, a user may upload a VCF file with SNPs of interest and return a list of genes that are mapped to the rsIDs provided by the user. The gene list provides information on each gene, including any enhancers that were linked according to PEREGRINE data. The user may alternatively wish to map the SNPs of interest directly to any PEREGRINE enhancers, which will also yield a list of genes that the mapped enhancers were linked to. The cell type-specific scores for these links are available in four cell lines available for download from the PEREGRINE website, allowing the user to select the cell type-specific score for the cell line most relevant to their research. For instance, researchers interested in colorectal cancer (CRC) may be interested in rs58920878, an SNP shown to be associated with increased disease risk (OR: 1.49, *p* = 0.0035)^27^. This SNP maps to just one enhancer in the PEREGRINE set, EH37E0467415, located in the intron of *SMAD7*. SMAD7 is a negative feedback regulator of the TGFβ signaling pathway, a pathway containing multiple genes known to be involved in the progression to CRC^28^. Changes in *SMAD7* expression levels have also been shown to influence the progression of CRC^29^. Further, the silencing of *SMAD7* using antisense RNA inhibits the proliferation of CRC cell lines both in vitro and in vivo after transplantation into immunodeficient mice^30^. The EH37E0467415 enhancer contains this SNP. The *Z*-score for the EH37E0467415-*SMAD7* enhancer-gene link in colorectal carcinoma cell line HCT116 is a formidable 7.55, a *Z*-score corresponding to the top 0.01% of possible enhancer-gene pairs in this cell type. Indeed, four functional SNPs (rs6507874, rs6507875, rs8085824, and rs58920878) contained within this enhancer have demonstrated allele-specific enhancer activity in HCT116, correlating with increased expression of *SMAD7* in normal colon epithelial tissues, and were located within sequences that bound to nuclear proteins from CRC cell lines in an allele-specific manner in electrophoretic mobility shift assays^31^. This enhancer-gene link scored far lower in other cell lines (HepG2 *Z*-score: 1.13, K562 *Z*-score: 4.12, MCF7 *Z*-score: −0.30), underscoring the cell-specific nature of this method. The high *Z*-score in K562 cells suggests that there could be a regulatory relationship between EH37E0467415-*SMAD7* in leukemia cells.

As another example, cyclin D1 (*CCND1*) is an important oncogene that is vital for cell-cycle progression and is thought to have an important role in breast cancer and other tumors. It is overexpressed in over 50% of breast cancer tumors^32^. The enhancer EH37E0225350 is located 126 kb upstream of *CCND1* in an intergenic region. The cell type-specific *Z*-score for the EH37E0225350-*CCND1* enhancer-gene link is 8.29 (corresponding to the top 0.14% of all possible enhancer-gene links) in the human adenocarcinoma cell line MCF7 which is often used to study breast cancer. This enhancer is located in a hotspot for ERα binding in MCF7 cells and interacts with the promoter of *CCND1*^33^. MCF7 cells treated with CRISPR-mediated deletion of this enhancer showed dramatically reduced eRNA expression and complete abolishment of *CCND1* mRNA activation^34^. *CCND1* and EH37E0225350 endogenous expression was shown to be dependent on estrogen signaling, indicating that the active EH37E0225350 enhancer may be necessary for the activation of *CCND1* expression by estrogen in breast cancer cells^34^. Notably, the EH37E0225350-*CCND1* enhancer-gene link scored much lower in other cell lines unrelated to breast cancer, such as HepG2 (*Z*-score: -0.30), HCT116 (*Z*-score: 3.07), and K562 (*Z*-score: −0.20), suggesting that the model has good ability to discriminate between different cellular environments for the same enhancer-gene pair.

*CYP2D6* encodes a member of the cytochrome P450 superfamily of enzymes and is believed to be involved in the metabolism of 25% of commonly prescribed drugs^35^. This gene is highly polymorphic within the human population and mostly expressed in the liver^36^. Allelic differences and copy number variations result in phenotypic changes to the ability to metabolize CYP2D6’s substrates, which characterize an individual’s metabolizer status anywhere from poor to ultrarapid^37^. Regulatory polymorphisms have also been shown to alter CYP2D6 expression at least twofold^38^. A distant downstream enhancer located in the intron of *WBP2NL*, EH37E0634729, was recorded in PEREGRINE as linked to *CYP2D6* in the previous analysis. This analysis attributed this enhancer-gene link a *Z*-score of 4.36 in the human hepatocyte carcinoma cell line, HepG2, putting the EH37E0634729-*CYP2D6* enhancer-gene link in the top 1.2% of scores for possible enhancer-gene pairs in that cell type. The enhancer EH37E0634729 contains rs5758550, an SNP shown to physically interact with the *CYP2D6* promoter and correlate with an increase in CYP2D6 in a pediatric cohort of individuals. Allelic mRNA expression analysis identified two SNPs in perfect linkage equilibrium, rs5758550/rs133333, which fully accounted for increased *CYP2D6* mRNA expression observed in livers^38^. Furthermore, CRISPR-mediated genome editing in HepG2 cells targeting putative enhancer regions in the ±2 kb area surrounding rs5758550 demonstrated a 70% decrease in *CYP2D6* mRNA expression, but “only upon deletion of the rs5758550 region”^39^. Interestingly, the EH37E0634729-*CYP2D6* enhancer-gene link does not score highly in other cell lines unrelated to the liver, such as HCT116 (*Z*-score: 0.25), K562 (*Z*-score: 0.64), and MCF7 (*Z*-score: 0.62).

### Comparison with other methods

PEACOCK predictions scored using the final model were compared with other available approaches, including a random classifier, distance-only model, and previously published predictions from the Activity-By-Contact (ABC) model, GeneHancer, and TargetFinder (Table 4). PEACOCK’s final model generates AUPRCs much higher than the random classifier and distance-only models on all available test datasets. PEACOCK also outperforms GeneHancer (AUPRC = 0.04), TargetFinder (AUPRC = 0.04), and ABC (AUPRC = 0.47). While PEACOCK usually outperforms other approaches, it lends value in more ways than just its predictive performance.

PEACOCK offers scores across the genome between all possible combinations of enhancers and genes within 1 Mb apart. It also offers these scores in the context of their cell-specific distribution in terms of both the number of standard distributions from the mean (*Z*-scores) and percentiles (*F*(scores)). Additionally, PEACOCK has been integrated into the PANTHER database, a resource already well-known to investigators who may lack the computational or statistical skillsets to utilize other predictive resources. Not only does PANTHER make the PEACOCK data more accessible to bench users, it also provides an extensive framework of biological context for enhancers and their linked genes by facilitating pathway analyses and other valuable annotations through its online suite of tools.

### Score interpretation

Each potential enhancer-gene regulatory link is provided a cell type-specific score by the final model. Although this score is within the [0,1] interval, it should not be interpreted as a probability. In a setting where the positive and negative classes are sampled in proportion to their frequency among all cis gene-enhancer pairs, the raw score generated by a probabilistic classification algorithm can be interpreted (possibly after calibration) as the probability of belonging to the positive class^26,39^. However, in our setting, the positive class and negative classes in the training sets are not necessarily proportional to their true frequencies (which are not known). Therefore, a higher raw score indicates a higher chance of belonging to the positive class, but not quantitatively as the probability of belonging to the positive class.

A probability has an absolute interpretation regardless of cell type environment. However, the cell type-specific scores are relative only with regard to the enhancer-gene link scores from the same cell type, and not to the enhancer-gene link scores for another cell type. Each cell type yields its own score distribution, which makes it inappropriate to consider the same classification cutoff for scores generated with different cell type-specific data. A cell type-specific score of 0.42 may be low in one cell type’s scoring distribution, but quite high for another cell type distribution. Therefore, it is not suitable to compare raw cell type-specific scores outside of the same cell type. Instead, the *Z*-score (a measure of how many standard deviations a score is from the mean of all scores in that cell type) or the *F*(score) (the percentile of the score out of all the scores in that cell type) of the cell type-specific scores form a better basis for comparing the confidence of predicted enhancer-gene regulatory links scored in different cell types. The *Z*-score and *F*(score) are accordingly provided for every enhancer-gene pair to make the scoring system more interpretable for the investigator.

### Systems biology implications

In an effort to contextualize the regulatory landscape of the cells scored in PEACOCK, some global analyses were conducted. Although the user can select any cutoff value that they want, enhancer-gene links with *F*(score) of no less than 0.95 (corresponding to the top 5% highest scoring enhancer-gene links in each cell line) were used to generate the statistics in Table 5.

Table 5 shows some of the most common points of interest when it comes to summarizing the regulatory landscape, such as how many enhancers regulate more than one gene, how many genes are regulated by more than one enhancer, and which enhancer or gene has the most regulatory partners. With the exception of the proportion of enhancers linked to more than one gene in HCT116 (noticeably lower), results seem to be consistent across cell types for these metrics. Interestingly, although almost all genes are linked to more than one enhancer, only around 28% of enhancers were linked to more than one gene. This could be accounted for by the fact that enhancers greatly outnumber genes, or by the possibility that most enhancers tend to regulate only one gene in each cell type. These results represent only a tiny sliver of the potential statistical analyses that PEACOCK scores can facilitate.

Future work will include investigations into more complex questions, such as how many of these highly scoring enhancer-gene regulatory predictions assemble into cis-regulatory modules (CRM) or complexes involving combinatorial regulation by more than one transcription factor. We also plan to analyze what certain additional features would bring to the model. For example, using the expression of the gene in the cell line as a feature may allow the final model to be reduced to fewer features. This could be beneficial in cell lines where gene expression data is available, but data for some of the other features are not. Exploratory analysis is also planned to investigate if adding a distance feature (genomic distance between enhancers and genes) can add predictive value.

### Perspectives

PEACOCK is a simple yet effective approach to providing cell type-specific scores to predicted enhancer-gene regulatory links across many widely studied cell lines. It requires the data from only a few common assays to generate features for all possible enhancer-gene links made up of enhancers and genes located <1 Mb apart. These scores provide an avenue for investigators to evaluate predicted enhancer-gene links from any database or collection of enhancer-gene predictions in cell types of interest. Although enhancer-gene prediction databases such as PEREGRINE are based on experimental evidence, it is clear, based on the distribution of the cell type-specific scores for all links predicted in PEREGRINE (Fig. 3) that many of the enhancer-gene links are likely not active in certain cell types, demonstrating the critical need for cell type-specific scoring when incorporating predicted enhancer-gene regulatory relationships for any application. By utilizing PEACOCK scores for cell types of interest, investigators will be able to contextualize predicted enhancer-gene regulatory relationships in disease-relevant settings.

In conclusion, enhancers play a vital role in orchestrating the extremely complicated system of gene expression necessary for human development and the continued maintenance of our differentiated cell populations. When normal enhancer regulation of genes is disrupted, the disease can arise. In fact, enhancer dysregulation has been associated with many diseases, including many types of cancer. Despite their importance, relatively few cell type-specific regulatory relationships between enhancers and their target genes have been identified. There have been many efforts at high throughput predictions of enhancer-gene regulatory links, but a statistically validated scoring system based on gold standard data in a cell type-specific context has been difficult to accomplish. Here we described the basis for attributing cell type-specific scores for over 17 million enhancer-gene pairs generated by a final model using the k-nearest neighbors algorithm trained on experimentally validated enhancer-gene links curated from peer-reviewed scientific articles (Supplementary Figure 17). For cell types missing P300 ChIP-seq data, cell type-specific scores were generated by a support vector machine alternate final model. These scores are available in four cell types, accessible in bulk download format from www.peregrineproj.org. Enhancer-gene links previously predicted in the PEREGRINE database and incorporated in the PANTHER Classification System (www.pantherdb.org) for gene and variant search also have cell type-specific scores attributed to them in the same cell types on the PANTHER platform. These cell type-specific scores will allow researchers to evaluate the quality of predicted enhancer-gene links in a more systematic way and to better harness the knowledge regarding enhancer-gene regulatory relationships. PEACOCK genome-wide scoring of enhancer-gene links in specific cellular settings provides the research community with an accessible tool for contextualizing disease-associated variants located within enhancers, facilitating the further investigation of the role of individual enhancers in many devastating genetic diseases.

## Methods

### Assembling the datasets

#### Positive class datasets

Enhancer-gene links comprising the positive training and test datasets (true active enhancer-gene links) were obtained from four different cancer cell lines. The datasets for HepG2 (liver) and HCT116 (colon) and MCF7 (breast) were obtained through careful PubMed searches. The search terms included the names of the cell lines and the word “enhancer” and terms related to enhancer-gene linking experiments, such as “CRISPR” or “luciferase.” The abstracts of the search results were then read to ascertain if the paper was relevant. If the paper seemed likely to include experimental evidence linking an enhancer to at least one target gene in the desired cell type, the paper and any necessary supplemental data were examined to determine if there was enough evidence (based on the criteria in Table 1) to include the enhancer-gene link in the curated list (Supplementary Table 1 of Supplementary Materials). The dataset for a third cell type, K562 (myelogenous leukemia), was obtained from published CRISPRi-FlowFISH experiments^40^. Positive enhancer-gene links were gathered from all results achieving statistical significance (*p* < 0.05) in experiments which perturbed enhancers within 450 kb of 30 genes and measured the effects on gene expression. Because there were two datasets in K562, this dataset is denoted as K562_1, and the next is denoted as K562_2. The K562_2 dataset was obtained from published CRISPRi experiments in K562 cells^40^. Positive enhancer-gene links were gathered from all results achieving statistical significance (FDR <0.05) in experiments where sgRNA-targeted enhancers located within 1 Mb of target genes *MYC* and *GATA1* were shown to significantly alter their gene expression.

The enhancer coordinates from the above datasets were mapped to the enhancers in the PEREGRINE enhancer set and the genes were recorded with their PANTHER long IDs. Only enhancer-gene links with protein-coding genes (recorded by their PANTHER long IDs) were considered for further analysis. PEREGRINE enhancers were mapped to an enhancer from the literature if there was at least a 33% overlap between their coordinates, which was calculated using bedtools^41^. For all enhancer-gene links that mapped to at least one PEREGRINE enhancer, the enhancer-gene link was recorded using the PEREGRINE enhancer ID and the gene’s PANTHER long ID. These enhancer-gene links made up the positive class of each dataset.

#### Negative class datasets

Enhancer-gene pairs comprising the negative training and test datasets were gathered based on the justification that physically inaccessible DNA could not contain enhancers and genes that were actively engaged in transcriptional regulatory activity. Although there is reason to believe that in *Drosophila*, some genes are active in the heterochromatic state^42^, we can find no evidence to show which genes this might be true for in human cells, if any. However, we recognize the possibility that such genes may exist and thereby erroneously be included in the negative set, although they would likely represent only a minute fraction of enhancer-gene pairs.

Additionally, there are areas of the genome for which euchromatin and heterochromatin-associated marks overlap, which have been documented to be enriched in imprinted genes^43^. This raises the possibility of heterochromatin marks in certain parts of the genome coinciding with active genes or enhancers, rendering ChIP-seq and other histone modification-targeting assays potentially problematic for determining which regions of the genome are inactive. FAIRE-seq and DNase-seq experiments assay for areas of the genome that are physically exposed and vulnerable to digestion, offering a potentially more exact representation of active versus inactive chromatin. Therefore, these DNA accessibility experiments were utilized for the construction of the negative set.

The bed files containing the peaks from ENCODE for DNase-seq and FAIRE-seq experiments were examined. Bedtools was used to determine which enhancers from the PEREGRINE set never overlapped with any of the peaks. Bedtools was also used to determine which genes (plus a 2 kb window on each end) had no overlap with any of the peaks. Enhancers or genes that met these requirements were considered inaccessible. All of the possible cis enhancer-gene links (enhancer-gene pairs where the enhancer and gene were located <1 Mb apart), which included either an inaccessible enhancer and/or an inaccessible gene, were labeled as negative.

**Fig. 4:**
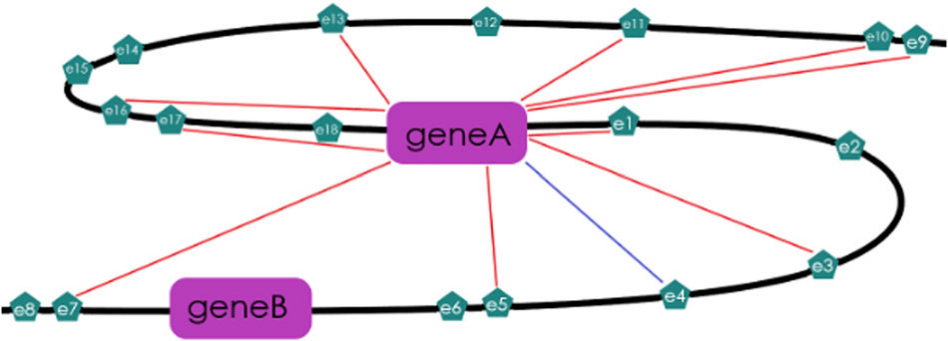
Negative enhancer-gene examples. The diagram illustrates how for each positive enhancer-gene example (gene A:e4 marked by a blue line), ten enhancers targeting the gene from the positive example (gene A) were randomly chosen from those in the complete set of negative examples to be included in the dataset (Ten enhancers connected to gene A by a red line). This was done so that the negative examples would come from a cellular environment similar to the positive examples. This was only performed for the HepG2, HCT116, and MCF7 datasets. The K562_1 and K562_2 datasets are from two different CRISPR datasets that reported negative and positive examples within the same ~1 Mb long region. Therefore, the experimental design for these two datasets ensured that the negative and positive examples came from the same genomic regions.

To generate the most useful training and test datasets, the negative class was sampled to match the genomic local environment of the positive class observations, reducing the chances of differences in features being the result of factors pertaining to anything other than the presence or absence of an active regulatory relationship between an enhancer and gene. This was done by randomly sampling the portion of the negative class, including cis enhancer-gene pairs targeting the genes found in enhancer-gene links in the positive set. Negative enhancer-gene pairs included in the training and test sets were required to target a gene that was found in a positive enhancer-gene link in the same cell type (Fig. 4). For each gene found in any enhancer-gene links in the positive class, 30 negative enhancer-gene pairs targeting that same gene were randomly sampled from total the negative sets for HepG2, HCT116, and MCF7 to comprise the negative class. For the K562_1 dataset, 400 enhancer-gene pairs were selected that were found to be inaccessible and failed to achieve statistical significance from CRISPR experiments (*p* > 0.10) which examined the effect that enhancer deletion had on previously selected nearby genes’ expression levels. For the K562_2 dataset, 1,303 enhancer-gene pairs were selected that failed to achieve statistical significance from CRISPRi experiments (FDR > 0.05) which examined the effect that enhancer perturbation had on nearby *MYC* or *GATA1* expression levels. It was then confirmed that none of them were in the positive set. These enhancer-gene pairs make up the final negative class instances in each dataset.

#### Combining the positive and negative classes into datasets

Negative examples were combined with positive examples to create each training or testing dataset in each cell line. Due to the fact that positive observations were so hard to come by (via a very time-consuming and labor-intensive literature search), we did not have as many as we would wish for. However, since negative observations were very easy to come by due to the fact that they were derived via DNA accessibility assays, we were not restricted in how many could be included in the training and testing datasets. The justification for including more negatives than positives was that even though it created a class imbalance, it should at least still give the algorithm more data to train on and, thereby, hopefully result in a better model. We are planning to investigate the use of SMOTE to address the class imbalance issue, but in the meantime, we believe that by reporting metrics that place more importance on the rare positive class (AUPRC instead of AUC, for example) we are providing an honest estimate of model performance.

#### Joint training datasets

Joint training datasets were assembled using enhancer-gene links from more than one cell type. Positively classed enhancer-gene links from multiple cell types were combined to make the positive class of enhancer-gene links for joint training sets. The negative class for joint training sets was assembled in the same way.

### Features

Features of an enhancer-gene pair include traits that are characteristic of the enhancer, the gene, and the enhancer and the gene simultaneously. Features which are classically relevant to enhancer activity on target genes were considered for establishing a feature set for enhancer-gene link data. There are nine main features in each cell type-specific dataset of enhancer-gene pairs. They are described in Table 2. All continuous variables were subsequently standardized (rescaled to have a mean of zero and a standard deviation of one). Since the eQTL features are based on GTEx’s cis-eQTL datasets, only cis enhancer-gene pairs were included in the dataset, as defined by GTEx, which was 1 Mb upstream or downstream of each gene’s transcription start site. The bedtools window command was used to gather all enhancers within 1 Mb of each gene’s transcription start site. These 17,354,145 cis enhancer-gene pairs were then attributed features based on the datasets described in Table 2.

The nearest gene was recorded as a binary value because we were interested in how often the nearest gene is the target gene of an enhancer, rather than incorporating the information conveyed in the genomic distance as a feature since, in some cases, the nearest gene may be much closer or farther away than the nearest gene for another enhancer. For other features, the decision to be reported as binary vs continuous was simply based on how the data was reported from the primary source it was collected from. For example, some ChIP-seq-based features are reported as binary because we opted to use data from ENCODE’s Registry of candidate cis-Regulatory Elements (cCREs) where available, which reported a list of enhancers with “high” peak levels for certain epigenomic signatures associated with active enhancers. This was also true for H3K4me3 peaks over promoters. ENCODE reported that as a binary result, so it was used as a binary feature in these analyses. In the case of H3K4me1 peaks over enhancers and H3K27ac over promoters, this was not available in the cCREs data, so ChIP-seq peak data reported from ENCODE was reported as a continuous variable.

Interaction terms were created between the features that were only for enhancers and only for promoters (Table 3). This was in an effort to create features in the model that represented the entire enhancer-gene link, rather than just the enhancer or just the gene. Interaction terms were also considered that were the result of an interaction effect—the estimate for a feature’s effect differing by levels of another feature in the model. This was clearly shown by eQTL * eQTL average absolute coefficient (the interaction of the eQTL combined Z-statistic, a measure of significance, and the average of the absolute values of the eQTL coefficients, a measure of effect size). Interactions between H3K4me1 and H3K27ac, two active enhancer marks, were also examined.

### Model selection and prediction performance

To select a predictive model that would be appropriate for this application (a modestly sized dataset with a binary outcome, relatively few features, and large class imbalance), the prediction performance of each model with all features was examined. Random forest, flexible discriminant analysis, linear discriminant analysis, gradient boosting machines, ridge regression, k-nearest neighbors, and support vector machines with Gaussian radial, polynomial, linear, hyperbolic tangent, Laplace radial, Bessel, and ANOVA radial basis kernels were all evaluated in terms of the AUPRC and AUC values they achieved on MCF7 and K562_2 test sets. Models were also evaluated on their prediction performance using any of the HepG2, HCT116, or K562_1 datasets that did not serve as training data for that model (i.e., a model trained on HepG2 data could be tested on the HCT116 dataset, the K562_1 dataset, the K562_2 dataset, or the MCF7 dataset.). No model was ever evaluated using test data containing observations that were used in the training dataset for that model. Default values were used for hyperparameters for models where hyperparameters exist.

To select the final model used for all enhancer-gene link scoring, the sum of the minimum AUPRC and the mean AUPRC across all tests was calculated to determine the highest-performing model, which also performed consistently well across test sets. For each training set, the best model was designated as the model with the highest sum of the minimum AUPRC and the mean AUPRC across validation sets. Models were disqualified if the minimum AUPRC was <0.10. Among the remaining best models from each training set (mean AUPRC: 0.55–0.64), the model with the highest mean AUC (0.93) was selected as the best-performing full model. Feature selection was then performed on this model by dropping each feature one by one and evaluating the AUPRC in the smaller model. The model performed slightly better without Feature 11 (Supplementary Table 16). Further dropping of features was not found to increase prediction performance (Supplementary Table 17). This was used as the final model for scoring new data (mean AUPRC: 0.65).

### Scoring new enhancer-gene links

All cis enhancer-gene pairs (*n* = 17,354,145) for which complete features data was available were scored with the HepG2 + K562_1-trained final model in a cell type-specific manner. All cis enhancer-gene pairs in cell types (e.g., HCT116) missing only the P300 feature were scored with the K562_1-trained final model in a cell type-specific manner. Of these, the scores of all cis PEREGRINE enhancer-gene links (*n* = 880,946) were examined. Two-sample KS tests were used to evaluate the difference in distributions of the scores of PEREGRINE enhancer-gene links compared to all cis enhancer-gene pairs not reported in PEREGRINE for all cell types.

### Comparison with other methods

The performance of the random classifier was measured by calculating the proportion of positive links within each dataset, which is equivalent to the AUPRC of a random classifier. The dataset used for this calculation appears at the head of each column in Table 4. A distance-only model was trained in each of the HepG2, HCT116, and K562_1 datasets. Each of those models was then tested on the remaining datasets and the AUPRC was calculated for each (Supplementary Tables 13–15). The distance between the enhancer and the gene was calculated by subtracting the midpoint of the enhancer from the midpoint of the gene in each link. A set of models using the absolute value of the distance between the gene and enhancer was also evaluated but did not yield any interesting differences (Supplementary Tables 13–15). The AUPRC reported in Table 4 for each test set is the highest AUPRC among all trained distance-only models, so comparisons would be as favorable toward distance-only models as possible. Models trained on the HepG2 dataset delivered the highest AUPRCs when tested on the HCT116, K562_1, and MCF7 datasets. Models trained on the HCT116 dataset delivered the highest AUPRCs when tested on the HepG2 and K562_2 datasets. A complete breakdown of all testing/training combinations can be found in Supplementary Tables 13–15.

To make comparisons between PEACOCK and other published methods fair, test datasets were filtered to include only observations that were predicted in both PEACOCK and the published method it was being compared against. Since each resource used a different set of enhancers, bedtools was used to evaluate the overlap between enhancers in both sets. Enhancers from existing resources found to overlap at least 33% with enhancers used by PEACOCK were deemed comparable. The same test dataset was then used to calculate the AUPRC for both PEACOCK and the published method being compared to PEACOCK. The PRC for both were plotted together for comparison (Supplementary Figs. 8–10). PEACOCK was compared separately to predictions generated by the ABC model, GeneHancer, and TargetFinder.

### Systems biology implications

Enhancer-gene links with *F*(score) of no less than 0.95 (corresponding to the top 5% highest scoring enhancer-gene links in each cell line) were taken as a subset of all 17 M scored enhancer-gene links in each cell line. For each of these subsets, genes that appear in more than one link and enhancers that appear in more than one link were tallied and entered as a percentage of the total number of genes and enhancers appearing in the subset. Additionally, the number of enhancers linked were tallied for each gene and sorted in order to obtain the gene with the largest number of linked enhancers. The enhancer linked to the most genes was derived analogously.

### Reporting summary

Further information on research design is available in the Nature Research Reporting Summary linked to this article.

## Supplementary information

Supplemental Materials

Reporting Summary

## Data Availability

The complete set of scored cis enhancer-gene pairs for all cell types are available in bulk data download files on the PEREGRINE website (www.peregrineproj.org). Cell type-specific scores for PEREGRINE enhancer-gene links were also integrated into the PANTHER web interface for querying PEREGRINE enhancer-gene link information (www.pantherdb.org).

## Code availability

The GitHub for this project, especially for training/testing datasets and pipelines, is available at https://github.com/USCbiostats/PEACOCK.
