# Supplementary material for: PEACOCK: a machine learning approach to assess the validity of cell type-specific enhancer-gene regulatory relationships
Source: NPJ Syst Biol Appl. 2023 Apr 3;9:9. doi: 10.1038/s41540-023-00270-z (PMC10070356; doi:10.1038/s41540-023-00270-z)

Supplementary Materials

Mills et. al.

Supplementary Tables.

Supplementary Table 1. Summary of enhancer-gene regulatory links curated from the scientific literature. The data were collected from 56 scientific paper with a total of 133 possible links. 93 of them are found in the PEREGRINE database, and are used as positive class for the ML training. 15 of them involve genes not found in the PANTHER database, and another 25 of them involve enhancers not found in the PEREGRINE database. Therefore, they are not used in the project.
The table is an Excel spreadsheet in a separate file.

Tissue/cell type	enhancer	gene	enhancer_chr	enhancer_start (hg19)	enhancer_end (hg19)	hg19	PMID
HCT-116	EH37E0652188	HUMAN|HGNC=7127|UniProtKB=P40692	chr3	37000088	37000663		29898989
HCT-116	EH37E0797418	HUMAN|HGNC=17260|UniProtKB=O95379	chr5	118659237	118659697		27834950
HCT-116	EH37E0797419	HUMAN|HGNC=17260|UniProtKB=O95379	chr5	118659857	118659949		27834950
HCT-116	53836	HUMAN|HGNC=10472|UniProtKB=Q13950	chr6	45413515	45413853		25769725
HCT-116	EH37E0843017	HUMAN|HGNC=10472|UniProtKB=Q13950	chr6	45413528	45414023		25769725
HCT-116	EH37E0978969	HUMAN|HGNC=7553|UniProtKB=P01106	chr8	128753818	128756102		18852287, 19966299, 28390865
HCT-116	58797	HUMAN|HGNC=7553|UniProtKB=P01106	chr8	128227053	128227754		26983878, 25801169, 19561607
HCT-116	EH37E0978690	HUMAN|HGNC=7553|UniProtKB=P01106	chr8	128227203	128227889		26983878, 25801169, 19561607
HCT-116	EH37E0978696	HUMAN|HGNC=7553|UniProtKB=P01106	chr8	128234839	128235525		26983878, 25801169, 19561607
HCT-116	EH37E0978711	HUMAN|HGNC=7553|UniProtKB=P01106	chr8	128259126	128260219		26983878, 25801169, 19561607
HCT-116	58806	HUMAN|HGNC=7553|UniProtKB=P01106	chr8	128259400	128259918		26983878, 25801169, 19561607
HCT-116	EH37E0978726	HUMAN|HGNC=7553|UniProtKB=P01106	chr8	128276753	128279405		26983878, 25801169, 19561607
HCT-116	58831	HUMAN|HGNC=7553|UniProtKB=P01106	chr8	128412842	128413241		26743005
HCT-116	EH37E0978793	HUMAN|HGNC=7553|UniProtKB=P01106	chr8	128413021	128413974		26743005
HCT-116	58832	HUMAN|HGNC=7553|UniProtKB=P01106	chr8	128413471	128413778		26743005
HCT-116	EH37E0978794	HUMAN|HGNC=7553|UniProtKB=P01106	chr8	128414166	128414499		26743005
HCT-116	EH37E0978795	HUMAN|HGNC=7553|UniProtKB=P01106	chr8	128414726	128415188		26743005
HCT-116	58831	HUMAN|HGNC=24166|UniProtKB=Q96KN1	chr8	128412842	128413241		26743005
HCT-116	EH37E0978793	HUMAN|HGNC=24166|UniProtKB=Q96KN1	chr8	128413021	128413974		26743005
HCT-116	58832	HUMAN|HGNC=24166|UniProtKB=Q96KN1	chr8	128413471	128413778		26743005
HCT-116	EH37E0978794	HUMAN|HGNC=24166|UniProtKB=Q96KN1	chr8	128414166	128414499		26743005
HCT-116	EH37E0978795	HUMAN|HGNC=24166|UniProtKB=Q96KN1	chr8	128414726	128415188		26743005
HCT-116	65512	HUMAN|HGNC=11641|UniProtKB=Q9NQB0	chr10	114757704	114758656		26845344
HCT-116	EH37E0194424	HUMAN|HGNC=11641|UniProtKB=Q9NQB0	chr10	114758265	114758616		26845344
HCT-116	EH37E0222066	HUMAN|HGNC=11026|UniProtKB=P08195	chr11	62617659	62617901		28825722
HCT-116	EH37E0222067	HUMAN|HGNC=11026|UniProtKB=P08195	chr11	62617902	62618012		28825722
HCT-116	EH37E0222068	HUMAN|HGNC=11026|UniProtKB=P08195	chr11	62618016	62618271		28825722
HCT-116	71632	HUMAN|HGNC=11026|UniProtKB=P08195	chr11	62618001	62618200		28825722
HCT-116	EH37E0239233	HUMAN|HGNC=30527|UniProtKB=Q8IXP5	chr11	111153396	111153799		24256810
HCT-116	EH37E0239234	HUMAN|HGNC=30527|UniProtKB=Q8IXP5	chr11	111153969	111154650		24256810
HCT-116	EH37E0239233	HUMAN|HGNC=33789|UniProtKB=Q6ZS62	chr11	111153396	111153799		24256810
HCT-116	EH37E0239234	HUMAN|HGNC=33789|UniProtKB=Q6ZS62	chr11	111153969	111154650		24256810
HCT-116	EH37E0239233	HUMAN|HGNC=26978|UniProtKB=A8K830	chr11	111153396	111153799		24256810
HCT-116	EH37E0239234	HUMAN|HGNC=26978|UniProtKB=A8K830	chr11	111153969	111154650		24256810
HCT-116	12808	HUMAN|HGNC=3495|UniProtKB=P41212	chr12	12009987	12010279		27145994
HCT-116	EH37E0254775	HUMAN|HGNC=3495|UniProtKB=P41212	chr12	12009738	12010385		27145994
HCT-116	EH37E0284634	HUMAN|HGNC=25645|UniProtKB=Q8N682	chr12	102249052	102250057		17397945
HCT-116	EH37E0440332	HUMAN|HGNC=18505|UniProtKB=Q68DV7	chr17	56468279	56468541		29021137, 24466159
HCT-116	EH37E0440333	HUMAN|HGNC=18505|UniProtKB=Q68DV7	chr17	56468773	56469278		29021137, 24466159
HCT-116	EH37E0440334	HUMAN|HGNC=18505|UniProtKB=Q68DV7	chr17	56471920	56472335		29021137, 24466159
HCT-116	25114	HUMAN|HGNC=18505|UniProtKB=Q68DV7	chr17	56468859	56469318		29021137, 24466159
HCT-116	27256	HUMAN|HGNC=6773|UniProtKB=O15105	chr18	46449093	46449527		25375357
HCT-116	EH37E0467415	HUMAN|HGNC=6773|UniProtKB=O15105	chr18	46448965	46449654		25375357
HepG2	28137	HUMAN|HGNC=6547|UniProtKB=P01130	chr19	11202212	11202837		27973560
HepG2	29328	HUMAN|HGNC=613|UniProtKB=P02649	chr19	45427550	45427884		7681840
HepG2	29330	HUMAN|HGNC=18001|UniProtKB=O96008	chr19	45454604	45454881		22089642
HepG2	36505	HUMAN|HGNC=5024|UniProtKB=P41235	chr20	43023129	43023488		26232425, 12504020
HepG2	57973	HUMAN|HGNC=2637|UniProtKB=P08684	chr7	99389389	99389610		10570062
HepG2	67975	HUMAN|HGNC=4124|UniProtKB=Q10471	chr1	230296401	230297600		26637976
HepG2	78982	HUMAN|HGNC=18001|UniProtKB=O96008	chr19	45454401	45455001		22089642
HepG2	86759	HUMAN|HGNC=74|UniProtKB=Q9UNQ0	chr4	89028600	89029201		27856528
HepG2	EH37E0113574	HUMAN|HGNC=8821|UniProtKB=Q5T2W1	chr1	145722958	145723117		30124855
HepG2	EH37E0113575	HUMAN|HGNC=8821|UniProtKB=Q5T2W1	chr1	145723320	145723991		30124855
HepG2	EH37E0145522	HUMAN|HGNC=4124|UniProtKB=Q10471	chr1	230294315	230295128		26637976, 26817450
HepG2	EH37E0194449	HUMAN|HGNC=11641|UniProtKB=Q9NQB0	chr10	114785306	114785388		29331016
HepG2	EH37E0194450	HUMAN|HGNC=11641|UniProtKB=Q9NQB0	chr10	114785391	114785571		29331016
HepG2	EH37E0492388	HUMAN|HGNC=613|UniProtKB=P02649	chr19	45427415	45427868		7681840
HepG2	EH37E0492392	HUMAN|HGNC=18001|UniProtKB=O96008	chr19	45454269	45455162		22089642
HepG2	EH37E0508180	HUMAN|HGNC=4196|UniProtKB=Q14397	chr2	27741177	27741349		28683826
HepG2	EH37E0634729	HUMAN|HGNC=2625|UniProtKB=P10635	chr22	42412183	42412615		23985325
HepG2	EH37E0634730	HUMAN|HGNC=2625|UniProtKB=P10635	chr22	42412710	42413102		25381333
HepG2	EH37E0734065	HUMAN|HGNC=74|UniProtKB=Q9UNQ0	chr4	88923747	88924836		28930109
HepG2	EH37E0734124	HUMAN|HGNC=74|UniProtKB=Q9UNQ0	chr4	89028360	89029535		27856528
HepG2	EH37E0901697	HUMAN|HGNC=5469|UniProtKB=P08833	chr7	45921319	45922372		29453285
HepG2	EH37E0917066	HUMAN|HGNC=2637|UniProtKB=P08684	chr7	99389279	99389989		10570062
HepG2	EH37E0917068	HUMAN|HGNC=2637|UniProtKB=P08684	chr7	99392310	99393275		14742674
MFC7	25079	HUMAN|HGNC=31350|UniProtKB=Q9NWM3	chr17	55977135	55977456		30145202
MFC7	39903	HUMAN|HGNC=1682|UniProtKB=Q08722	chr3	107705661	107706042		28378740
MFC7	39904	HUMAN|HGNC=1682|UniProtKB=Q08722	chr3	107706134	107706639		28378740
MFC7	39920	HUMAN|HGNC=1682|UniProtKB=Q08722	chr3	107850030	107850463		28378740
MFC7	53410	HUMAN|HGNC=1784|UniProtKB=P38936	chr6	36634782	36635224		26751173
MFC7	86758	HUMAN|HGNC=74|UniProtKB=Q9UNQ0	chr4	89026200	89026401		27856528
MFC7	EH37E0225350	HUMAN|HGNC=1582|UniProtKB=P24385	chr11	69329818	69330742		26751173

MFC7	EH37E0225351	HUMAN|HGNC=1582|UniProtKB=P24385	chr11	69330794	69331012		26751173
MFC7	EH37E0440131	HUMAN|HGNC=31350|UniProtKB=Q9NWM3	chr17	55977260	55977535		30145202
MFC7	EH37E0502078	HUMAN|HGNC=24885|UniProtKB=Q4ZG55	chr2	11638080	11638139		31905229
MFC7	EH37E0502079	HUMAN|HGNC=24885|UniProtKB=Q4ZG55	chr2	11638146	11638299		31905229
MFC7	EH37E0502080	HUMAN|HGNC=24885|UniProtKB=Q4ZG55	chr2	11638587	11639143		31905229
MFC7	EH37E0601829	HUMAN|HGNC=32594|UniProtKB=Q8TCU6	chr20	47480768	47481648		31372638
MFC7	EH37E0619581	HUMAN|HGNC=11755|UniProtKB=P04155	chr21	43795557	43795953		31905229
MFC7	EH37E0619581	HUMAN|HGNC=11756|UniProtKB=Q03403	chr21	43795557	43795953		31905229
MFC7	EH37E0619581	HUMAN|HGNC=11756|UniProtKB=Q03403	chr21	43795557	43795953		31905229
MFC7	EH37E0619582	HUMAN|HGNC=11755|UniProtKB=P04155	chr21	43796316	43797148		31905229
MFC7	EH37E0619582	HUMAN|HGNC=11757|UniProtKB=Q07654	chr21	43796316	43797148		31905229
MFC7	EH37E0619582	HUMAN|HGNC=11757|UniProtKB=Q07654	chr21	43796316	43797148		31905229
MFC7	EH37E0675037	HUMAN|HGNC=1682|UniProtKB=Q08722	chr3	107705245	107706641		28378740
MFC7	EH37E0675134	HUMAN|HGNC=1682|UniProtKB=Q08722	chr3	107849164	107849451		28378740
MFC7	EH37E0675135	HUMAN|HGNC=1682|UniProtKB=Q08722	chr3	107849460	107850783		28378740
MFC7	EH37E0734065	HUMAN|HGNC=74|UniProtKB=Q9UNQ0	chr4	88923747	88924836		27856528
MFC7	EH37E0734119	HUMAN|HGNC=74|UniProtKB=Q9UNQ0	chr4	89026095	89026375		27856528
MFC7	EH37E0734120	HUMAN|HGNC=74|UniProtKB=Q9UNQ0	chr4	89026446	89026647		27856528
MFC7	EH37E0734180	HUMAN|HGNC=74|UniProtKB=Q9UNQ0	chr4	89163314	89163772		27856528
MFC7	EH37E0838343	HUMAN|HGNC=1784|UniProtKB=P38936	chr6	36634770	36635337		26751173

links not involving PANTHER genes

HCT-116	58831	CCAT1	chr8	128412842	128413241	RNA gene	26743005
HCT-116	EH37E0978793	CCAT1	chr8	128413021	128413974	RNA gene	26743005
HCT-116	58832	CCAT1	chr8	128413471	128413778	RNA gene	26743005
HCT-116	EH37E0978794	CCAT1	chr8	128414166	128414499	RNA gene	26743005
HCT-116	EH37E0978795	CCAT1	chr8	128414726	128415188	RNA gene	26743005
HCT-116	58831	CASC8	chr8	128412842	128413241	RNA gene	26743005
HCT-116	EH37E0978793	CASC8	chr8	128413021	128413974	RNA gene	26743005
HCT-116	58832	CASC8	chr8	128413471	128413778	RNA gene	26743005
HCT-116	EH37E0978794	CASC8	chr8	128414166	128414499	RNA gene	26743005
HCT-116	EH37E0978795	CASC8	chr8	128414726	128415188	RNA gene	26743005
HCT-116	58831	PVT1	chr8	128412842	128413241	RNA gene	26743005
HCT-116	EH37E0978793	PVT1	chr8	128413021	128413974	RNA gene	26743005
HCT-116	58832	PVT1	chr8	128413471	128413778	RNA gene	26743005
HCT-116	EH37E0978794	PVT1	chr8	128414166	128414499	RNA gene	26743005
HCT-116	EH37E0978795	PVT1	chr8	128414726	128415188	RNA gene	26743005

links not involving PEREGRINE enhancers

HCT-116		HUMAN|HGNC=5010|UniProtKB=P17096	chr6	34203367	34203553		17510387
HCT-116		HUMAN|HGNC=7553|UniProtKB=P01106	chr8	128747285	128747872		18852287
HCT-116		HUMAN|HGNC=11920|UniProtKB=P25445	chr10	90751044	90751239		12700649
HCT-116		HUMAN|HGNC=16262|UniProtKB=P46937	chr11	101982740	101982819		22337891
HCT-116		HUMAN|HGNC=17855|UniProtKB=O94923	chr15	69451643	69451693		15853773
HCT-116		HUMAN|HGNC=18505|UniProtKB=Q68DV7	chr17	56497854	56498004		29021137
HCT-116		HUMAN|HGNC=904|UniProtKB=Q9Y2T1	chr17	63553694	63553803		28390865
HCT-116		HUMAN|HGNC=16076|UniProtKB=Q9BYG4	chr18	78018726	78018796		26658110
HCT-116		HUMAN|HGNC=12441|UniProtKB=P04818	chr18	657523	657764		20531375
HepG2		HUMAN|HGNC=74|UniProtKB=Q9UNQ0	chr4	89010953	89011437		27856528
HepG2		HUMAN|HGNC=74|UniProtKB=Q9UNQ0	chr4	89073171	89073422		27856528
HepG2		HUMAN|HGNC=74|UniProtKB=Q9UNQ0	chr4	89073197	89073197		28930109
HepG2		HUMAN|HGNC=74|UniProtKB=Q9UNQ0	chr4	89127612	89127877		27856528
HepG2		HUMAN|HGNC=74|UniProtKB=Q9UNQ0	chr4	89189503	89190458		27856528
HepG2		HUMAN|HGNC=12698|UniProtKB=P98155	chr9	2640759	2640759		30445632
HepG2		HUMAN|HGNC=1869|UniProtKB=P11597	chr16	56988044	56988044		26061659
HepG2		HUMAN|HGNC=1869|UniProtKB=P11597	chr16	56989590	56989590		26061659
HepG2		HUMAN|HGNC=23089|UniProtKB=Q86YT5	chr17	6618466	6618706		25628225
HepG2		HUMAN|HGNC=23089|UniProtKB=Q86YT5	chr17	6639150	6639424		25628225
HepG2		HUMAN|HGNC=4017|UniProtKB=P51993	chr19	5832773	5832773		28334792
HepG2		HUMAN|HGNC=6547|UniProtKB=P01130	chr19	11201124	11201124		27973560
HepG2		HUMAN|HGNC=2615|UniProtKB=P20813	chr19	40982528	40982938		12571232
HepG2		HUMAN|HGNC=2615|UniProtKB=P20813	chr19	41496857	41496873		26930610
HepG2		HUMAN|HGNC=18001|UniProtKB=O96008	chr19	45395619	45396665		22089642
HepG2		HUMAN|HGNC=18001|UniProtKB=O96008	chr19	45415640	45416478		22089642

Supplementary Table 2. Learners used to generate each model. This table provides the legend to abbreviations used in the subsequent supplemental tables due to limited space.

Model number	Learner
M1	Random forest
M2	Flexible discriminant analysis
M3	k-nearest neighbors
M4	Support Vector Machine (Gaussian kernel)
M5	Support Vector Machine (polynomial kernel)
M6	Support Vector Machine (linear kernel)
M7	Support Vector Machine (hyperbolic tangent kernel)
M8	Support Vector Machine (LaPlace kernel)
M9	Support Vector Machine (Bessel kernel)
M10	Support Vector Machine (ANOVA kernel)
M11	Linear discriminant analysis
M12	Gradient boosting machine
M13	Ridge regression

Supplementary Table 3. AUPRCs for models trained in HepG2 data using the algorithms in Table S1 and tested on the listed test datasets. K562_1 and K562_2 are two different datasets from two different publications, but both use K562 cells.

Training data: HepG2<br>AUPRC													
Test data	M1	M2	M3	M4	M5	M6	M7	M8	M9	M10	M11	M12	M13
MCF7	0.69<br>0.87	0.43<br>0.84	0.57<br>0.82	0.51<br>0.85	0.70<br>0.91	0.70<br>0.91	0.27<br>0.84	0.76<br>0.88	0.07<br>0.27	0.52<br>0.84	0.70<br>0.90	0.32<br>0.82	0.76<br>0.88
K562_2	0.23<br>0.97	0.10<br>0.64	0.37<br>0.91	0.24<br>0.98	0.37<br>0.95	0.37<br>0.95	0.06<br>0.95	0.62<br>0.86	0.00<br>0.20	0.12<br>0.96	0.35<br>0.95	0.03<br>0.67	0.35<br>0.98
K562_1	0.54<br>0.79	0.12<br>0.42	0.33<br>0.65	0.48<br>0.80	0.61<br>0.75	0.61<br>0.75	0.20<br>0.68	0.55<br>0.80	0.15<br>0.50	0.48<br>0.78	0.56<br>0.78	0.29<br>0.70	0.56<br>0.78

Supplementary Table 4. AUPRCs for models trained in HepG2 data using the algorithms in Table S1 and tested on the listed test datasets. In these models the P300 feature was not present. K562_1 and K562_2 are two different datasets from two different publications, but both use K562 cells.

Training data: HepG2 (no P300)<br>AUPRC													
Test data	M1	M2	M3	M4	M5	M6	M7	M8	M9	M10	M11	M12	M13
MCF7	0.36<br>0.83	0.06<br>0.32	0.27<br>0.71	0.49<br>0.85	0.38<br>0.86	0.38<br>0.86	0.13<br>0.69	0.43<br>0.86	0.06<br>0.25	0.26<br>0.81	0.26<br>0.68	0.41<br>0.82	0.58<br>0.85
K562_2	0.02<br>0.86	0.00<br>0.33	0.02<br>0.62	0.03<br>0.93	0.04<br>0.84	0.03<br>0.84	0.01<br>0.66	0.03<br>0.89	0.18<br>0.69	0.02<br>0.84	0.02<br>0.85	0.01<br>0.69	0.03<br>0.92
HCT116	0.28<br>0.78	0.07<br>0.40	0.19<br>0.70	0.27<br>0.67	0.35<br>0.76	0.35<br>0.76	0.14<br>0.62	0.34<br>0.75	0.07<br>0.33	0.31<br>0.78	0.35<br>0.79	0.41<br>0.85	0.48<br>0.87
K562_1	0.27<br>0.71	0.20<br>0.63	0.21<br>0.63	0.25<br>0.73	0.29<br>0.71	0.29<br>0.71	0.21<br>0.64	0.31<br>0.73	0.15<br>0.52	0.27<br>0.71	0.28<br>0.69	0.29<br>0.74	0.30<br>0.76

Supplementary Table 5. AUPRCs for models trained in HCT116 data using the algorithms in Table S1 and tested on the listed test datasets. In these models the P300 feature was not present because it was not available in HCT116. K562_1 and K562_2 are two different datasets from two different publications, but both use K562 cells.

Training data: HCT116 (no P300)<br>AUPRC													
Test data	M1	M2	M3	M4	M5	M6	M7	M8	M9	M10	M11	M12	M13
MCF7	0.31<br>0.87	0.13<br>0.47	0.09<br>0.57	0.22<br>0.79	0.32<br>0.67	0.32<br>0.67	0.17<br>0.51	0.45<br>0.84	0.10<br>0.43	0.42<br>0.67	0.27<br>0.66	0.37<br>0.81	0.56<br>0.87
K562_2	0.01<br>0.79	0.00<br>0.32	0.01<br>0.49	0.02<br>0.88	0.00<br>0.30	0.00<br>0.30	0.00<br>0.36	0.01<br>0.64	0.01<br>0.61	0.01<br>0.53	0.01<br>0.81	0.01<br>0.51	0.03<br>0.92
HepG2	0.14<br>0.80	0.05<br>0.36	0.07<br>0.59	0.12<br>0.73	0.18<br>0.65	0.18<br>0.65	0.14<br>0.48	0.17<br>0.78	0.07<br>0.47	0.12<br>0.69	0.16<br>0.63	0.17<br>0.76	0.27<br>0.89
K562_1	0.31<br>0.74	0.21<br>0.48	0.18<br>0.61	0.21<br>0.71	0.25<br>0.62	0.26<br>0.62	0.17<br>0.44	0.27<br>0.65	0.20<br>0.61	0.30<br>0.69	0.23<br>0.61	0.38<br>0.66	0.27<br>0.74

Supplementary Table 6. AUPRCs for models trained in K562_1 data using the algorithms in Table S1 and tested on the listed test datasets.

Training data: K562_1<br>AUPRC													
Test data	M1	M2	M3	M4	M5	M6	M7	M8	M9	M10	M11	M12	M13
MCF7	0.67<br>0.87	0.59<br>0.82	0.65<br>0.86	0.23<br>0.79	0.62<br>0.87	0.64<br>0.88	0.51<br>0.70	0.46<br>0.84	0.05<br>0.24	0.63<br>0.87	0.68<br>0.87	0.67<br>0.87	0.75<br>0.86
K562_2	0.23<br>0.99	0.09<br>0.98	0.45<br>0.99	0.33<br>0.97	0.37<br>0.98	0.38<br>0.99	0.03<br>0.87	0.46<br>1.00	0.13<br>0.82	0.08<br>0.98	0.35<br>0.97	0.07<br>0.97	0.36<br>0.99
HepG2	0.53<br>0.89	0.56<br>0.88	0.55<br>0.91	0.31<br>0.84	0.56<br>0.81	0.55<br>0.81	0.27<br>0.62	0.68<br>0.93	0.06<br>0.38	0.54<br>0.84	0.56<br>0.74	0.49<br>0.84	0.61<br>0.93

Supplementary Table 7. AUPRCs for models trained in K562_1 data using the algorithms in Table S1 and tested on the listed test datasets. In these models the P300 feature was not present.

Training data: K562_1 (no P300)<br>AUPRC													
Test data	M1	M2	M3	M4	M5	M6	M7	M8	M9	M10	M11	M12	M13
MCF7	0.48<br>0.84	0.41<br>0.83	0.23<br>0.75	0.25<br>0.79	0.36<br>0.83	0.30<br>0.80	0.09<br>0.51	0.26<br>0.81	0.19<br>0.54	0.55<br>0.85	0.35<br>0.84	0.54<br>0.87	0.32<br>0.81
K562_2	0.14<br>0.90	0.02<br>0.72	0.03<br>0.78	0.07<br>0.35	0.01<br>0.57	0.00<br>0.43	0.04<br>0.75	0.02<br>0.69	0.18<br>0.71	0.17<br>0.67	0.01<br>0.74	0.03<br>0.90	0.01<br>0.80
HepG2	0.24<br>0.87	0.28<br>0.84	0.35<br>0.90	0.25<br>0.83	0.36<br>0.81	0.33<br>0.77	0.09<br>0.49	0.40<br>0.91	0.09<br>0.51	0.36<br>0.84	0.39<br>0.89	0.24<br>0.74	0.35<br>0.91
HCT116	0.46<br>0.86	0.39<br>0.75	0.31<br>0.79	0.31<br>0.75	0.33<br>0.70	0.28<br>0.68	0.16<br>0.68	0.38<br>0.80	0.26<br>0.58	0.42<br>0.81	0.33<br>0.74	0.40<br>0.75	0.33<br>0.76

Supplementary Table 8. AUPRCs for models trained in HepG2+HCT116 data using the algorithms in Table S1 and tested on the listed test datasets. In these models the P300 feature was not present because it was not available in HCT116. K562_1 and K562_2 are two different datasets from two different publications, but both use K562 cells.

Training data: HepG2+HCT116 (no P300)<br>AUPRC													
Test data	M1	M2	M3	M4	M5	M6	M7	M8	M9	M10	M11	M12	M13
MCF7	0.43<br>0.85	0.17<br>0.67	0.16<br>0.70	0.41<br>0.86	0.30<br>0.74	0.30<br>0.75	0.15<br>0.64	0.48<br>0.85	0.41<br>0.74	0.46<br>0.80	0.48<br>0.86	0.46<br>0.87	0.55<br>0.87
K562_2	0.01<br>0.77	0.01<br>0.68	0.01<br>0.58	0.03<br>0.91	0.02<br>0.79	0.02<br>0.79	0.02<br>0.76	0.03<br>0.91	0.18<br>0.36	0.01<br>0.45	0.02<br>0.86	0.01<br>0.70	0.02<br>0.90
K562_1	0.27<br>0.75	0.23<br>0.63	0.25<br>0.65	0.32<br>0.76	0.28<br>0.71	0.28<br>0.71	0.25<br>0.69	0.37<br>0.78	0.22<br>0.57	0.30<br>0.75	0.28<br>0.73	0.27<br>0.70	0.29<br>0.77

Supplementary Table 9. AUPRCs for models trained in HepG2+K562_1 data using the algorithms in Table S1 and tested on the listed test datasets.

Training data: HepG2+K562_1<br>AUPRC													
Test data	M1	M2	M3	M4	M5	M6	M7	M8	M9	M10	M11	M12	M13
MCF7	0.69<br>0.86	0.60<br>0.86	0.70<br>0.87	0.45<br>0.87	0.70<br>0.85	0.68<br>0.89	0.37<br>0.66	0.62<br>0.91	0.05<br>0.17	0.41<br>0.80	0.76<br>0.91	0.69<br>0.90	0.77<br>0.89
K562_2	0.20<br>0.98	0.13<br>0.98	0.50<br>1.00	0.49<br>0.99	0.39<br>0.97	0.37<br>0.96	0.02<br>0.84	0.44<br>0.99	0.02<br>0.53	0.07<br>0.97	0.37<br>0.97	0.09<br>0.98	0.38<br>0.99

Supplementary Table 10. AUPRCs for models trained in HepG2+K562_1 data using the algorithms in Table S1 and tested on the listed test datasets. In these models the P300 feature was not present.

Training data: HepG2+K562_1 (no P300)<br>AUPRC													
Test data	M1	M2	M3	M4	M5	M6	M7	M8	M9	M10	M11	M12	M13
MCF7	0.46<br>0.84	0.36<br>0.85	0.38<br>0.75	0.37<br>0.73	0.52<br>0.90	0.50<br>0.89	0.14<br>0.60	0.48<br>0.89	0.22<br>0.68	0.32<br>0.80	0.43<br>0.88	0.54<br>0.88	0.53<br>0.86
K562_2	0.12<br>0.90	0.02<br>0.78	0.04<br>0.91	0.03<br>0.66	0.03<br>0.92	0.02<br>0.89	0.02<br>0.75	0.03<br>0.83	0.00<br>0.18	0.02<br>0.84	0.02<br>0.81	0.03<br>0.84	0.03<br>0.91
HCT116	0.33<br>0.81	0.44<br>0.76	0.32<br>0.78	0.38<br>0.69	0.38<br>0.78	0.39<br>0.78	0.12<br>0.58	0.41<br>0.82	0.32<br>0.72	0.42<br>0.82	0.42<br>0.78	0.50<br>0.83	0.46<br>0.84

Supplementary Table 11. AUPRCs for models trained in HCT116+K562_1 data using the algorithms in Table S1 and tested on the listed test datasets. In these models the P300 feature was not present because it was not available in HCT116.

Training data: HCT116+K562_1 (no P300)<br>AUPRC<br>AUC													
Test data	M1	M2	M3	M4	M5	M6	M7	M8	M9	M10	M11	M12	M13
MCF7	0.50<br>0.85	0.39<br>0.86	0.13<br>0.69	0.31<br>0.80	0.34<br>0.86	0.32<br>0.83	0.12<br>0.64	0.45<br>0.82	0.12<br>0.39	0.51<br>0.83	0.43<br>0.88	0.62<br>0.88	0.45<br>0.88
K562_2	0.11<br>0.90	0.03<br>0.79	0.04<br>0.92	0.19<br>0.48	0.01<br>0.57	0.00<br>0.44	0.01<br>0.71	0.19<br>0.78	0.06<br>0.52	0.18<br>0.68	0.02<br>0.88	0.02<br>0.80	0.03<br>0.93
HepG2	0.17<br>0.84	0.26<br>0.83	0.16<br>0.68	0.21<br>0.60	0.18<br>0.69	0.15<br>0.63	0.11<br>0.64	0.31<br>0.89	0.05<br>0.33	0.17<br>0.80	0.40<br>0.91	0.31<br>0.90	0.43<br>0.92

Supplementary Table 12. AUPRCs for models trained in HepG2+HCT116+K562_1 data using the algorithms in Table S1 and tested on the listed test datasets. In these models the P300 feature was not present because it was not available in HCT116.

Training data: HepG2+HCT116+K562_1 (no P300)<br>AUPRC													
Test data	M1	M2	M3	M4	M5	M6	M7	M8	M9	M10	M11	M12	M13
MCF7	0.50<br>0.85	0.49<br>0.85	0.14<br>0.67	0.29<br>0.77	0.09<br>0.43	0.06<br>0.38	0.16<br>0.66	0.48<br>0.86	0.41<br>0.74	0.39<br>0.83	0.47<br>0.89	0.61<br>0.88	0.49<br>0.88
K562_2	0.13<br>0.90	0.01<br>0.80	0.04<br>0.92	0.05<br>0.85	0.00<br>0.07	0.02<br>0.80	0.02<br>0.76	0.04<br>0.79	0.00<br>0.27	0.04<br>0.87	0.02<br>0.83	0.03<br>0.85	0.03<br>0.92

Supplementary Table 13. Distance-only models trained in HepG2. The top half of this table displays the AUPRC and AUC of a distance-only model trained on the HepG2 dataset and evaluated in the listed test datasets. The lower half of the table displays the AUPRC and AUC of a distance-only model trained on the HepG2 dataset and evaluated in the listed test datasets where the absolute value of the distance between the gene and enhancer is used.

Training data: HepG2 (distance-only model)<br>AUPRC<br>AUC													
Test data	M1	M2	M3	M4	M5	M6	M7	M8	M9	M10	M11	M12	M13
HCT116	0.26	0.43	0.25	0.17	0.09	0.09	0.39	0.22	0.25	0.29	0.09	0.39	0.38
	0.71	0.73	0.71	0.74	0.51	0.51	0.77	0.62	0.75	0.79	0.51	0.77	0.77
MCF7	0.19	0.36	0.22	0.26	0.08	0.08	0.30	0.23	0.05	0.38	0.08	0.26	0.29
	0.73	0.90	0.74	0.86	0.54	0.54	0.89	0.80	0.11	0.89	0.54	0.87	0.89
K562_1	0.31	0.46	0.36	0.29	0.14	0.14	0.53	0.31	0.25	0.26	0.14	0.41	0.55
	0.75	0.78	0.76	0.77	0.58	0.58	0.79	0.74	0.74	0.76	0.58	0.78	0.79
K562_2	0.01	0.03	0.01	0.00	0.00	0.00	0.03	0.01	0.01	0.01	0.00	0.01	0.01
	0.59	0.71	0.61	0.35	0.52	0.52	0.75	0.57	0.70	0.63	0.52	0.52	0.75
Training data: HepG2 (absolute value distance-only model)<br>AUPRC<br>AUC													
Test data	M1	M2	M3	M4	M5	M6	M7	M8	M9	M10	M11	M12	M13
HCT116	0.31	0.39	0.30	0.27	0.06	0.39	0.09	0.37	0.44	0.23	0.39	0.29	0.38
	0.72	0.76	0.72	0.68	0.23	0.77	0.55	0.67	0.77	0.77	0.77	0.74	0.77
MCF7	0.15	0.28	0.16	0.28	0.05	0.29	0.09	0.17	0.38	0.26	0.29	0.25	0.29
	0.65	0.87	0.66	0.86	0.11	0.89	0.60	0.65	0.90	0.86	0.89	0.88	0.89
K562_1	0.33	0.55	0.34	0.36	0.55	0.55	0.10	0.38	0.45	0.33	0.55	0.34	0.55
	0.77	0.79	0.77	0.77	0.79	0.79	0.37	0.73	0.79	0.79	0.79	0.78	0.79
K562_2	0.01	0.01	0.01	0.04	0.01	0.01	0.02	0.01	0.01	0.01	0.01	0.01	0.01
	0.68	0.59	0.68	0.60	0.75	0.75	0.73	0.54	0.75	0.58	0.75	0.63	0.75

Supplementary Table 14. Distance-only models trained in HCT116. The top half of this table displays the AUPRC and AUC of a distance-only model trained on the HCT116 dataset and evaluated in the listed test datasets. The lower half of the table displays the AUPRC and AUC of a distance-only model trained on the HCT116 dataset and evaluated in the listed test datasets where the absolute value of the distance between the gene and enhancer is used.

Training data: HCT116 (distance-only model)<br>AUPRC<br>AUC													
Test data	M1	M2	M3	M4	M5	M6	M7	M8	M9	M10	M11	M12	M13
HepG2	0.27	0.47	0.27	0.42	0.06	0.06	0.50	0.32	0.04	0.22	0.06	0.32	0.46
	0.78	0.88	0.78	0.82	0.49	0.49	0.96	0.78	0.35	0.90	0.49	0.86	0.96
MCF7	0.20	0.22	0.21	0.21	0.07	0.07	0.05	0.23	0.07	0.30	0.07	0.27	0.29
	0.70	0.72	0.70	0.66	0.46	0.46	0.10	0.62	0.41	0.84	0.46	0.79	0.89
K562_1	0.31	0.46	0.33	0.44	0.10	0.10	0.08	0.39	0.13	0.21	0.10	0.40	0.55
	0.70	0.80	0.70	0.80	0.42	0.42	0.22	0.72	0.55	0.71	0.42	0.75	0.79
K562_2	0.01	0.03	0.01	0.03	0.00	0.00	0.00	0.01	0.01	0.01	0.00	0.01	0.01
	0.56	0.42	0.58	0.58	0.52	0.48	0.25	0.56	0.60	0.47	0.48	0.50	0.75
Training data: HCT116 (absolute value distance-only model)<br>AUPRC<br>AUC													
Test data	M1	M2	M3	M4	M5	M6	M7	M8	M9	M10	M11	M12	M13
HepG2	0.35	0.38	0.41	0.33	0.47	0.47	0.05	0.33	0.44	0.43	0.47	0.56	0.47
	0.84	0.93	0.85	0.67	0.96	0.96	0.47	0.73	0.91	0.85	0.96	0.93	0.96
MCF7	0.19	0.23	0.22	0.16	0.29	0.29	0.08	0.27	0.21	0.15	0.29	0.28	0.29
	0.73	0.85	0.77	0.46	0.89	0.89	0.50	0.67	0.77	0.49	0.89	0.86	0.89
K562_1	0.35	0.54	0.43	0.54	0.55	0.55	0.18	0.43	0.55	0.56	0.55	0.48	0.55
	0.72	0.78	0.73	0.78	0.79	0.79	0.35	0.68	0.80	0.82	0.79	0.82	0.79
K562_2	0.01	0.01	0.01	0.01	0.01	0.01	0.00	0.01	0.01	0.01	0.01	0.01	0.01
	0.54	0.61	0.48	0.48	0.75	0.75	0.55	0.49	0.46	0.48	0.75	0.59	0.75

Supplementary Table 15. Distance-only models trained in K562_1. The top half of this table displays the AUPRC and AUC of a distance-only model trained on the K562_1 dataset and evaluated in the listed test datasets. The lower half of the table displays the AUPRC and AUC of a distance-only model trained on the K562_1 dataset and evaluated in the listed test datasets where the absolute value of the distance between the gene and enhancer is used.

Training data: K562_1 (distance-only model)<br>AUPRC<br>AUC													
Test data	M1	M2	M3	M4	M5	M6	M7	M8	M9	M10	M11	M12	M13
HepG2	0.30	0.39	0.31	0.40	0.06	0.06	0.45	0.30	0.06	0.44	0.06	0.37	0.46
	0.76	0.94	0.76	0.86	0.51	0.51	0.96	0.71	0.56	0.87	0.51	0.84	0.96
HCT116	0.24	0.25	0.24	0.35	0.09	0.09	0.34	0.36	0.18	0.23	0.09	0.39	0.40
	0.63	0.63	0.65	0.67	0.51	0.51	0.79	0.70	0.62	0.74	0.51	0.74	0.77
MCF7	0.12	0.23	0.14	0.14	0.08	0.07	0.32	0.13	0.09	0.18	0.08	0.17	0.29
	0.62	0.82	0.63	0.47	0.54	0.46	0.89	0.53	0.58	0.74	0.54	0.69	0.90
K562_2	0.03	0.01	0.02	0.01	0.00	0.00	0.01	0.02	0.00	0.01	0.00	0.01	0.01
	0.94	0.54	0.92	0.74	0.52	0.52	0.74	0.85	0.52	0.57	0.52	0.42	0.69
Training data: K562_1 (absolute value distance-only model)<br>AUPRC<br>AUC													
Test data	M1	M2	M3	M4	M5	M6	M7	M8	M9	M10	M11	M12	M13
HepG2	0.25	0.43	0.32	0.33	0.47	0.47	0.16	0.31	0.42	0.43	0.47	0.43	0.46
	0.77	0.86	0.78	0.69	0.96	0.96	0.85	0.71	0.91	0.85	0.96	0.94	0.96
HCT116	0.20	0.37	0.23	0.32	0.39	0.39	0.12	0.34	0.34	0.39	0.39	0.36	0.40
	0.65	0.72	0.65	0.61	0.77	0.77	0.63	0.61	0.77	0.77	0.77	0.70	0.78
MCF7	0.10	0.18	0.12	0.13	0.29	0.05	0.18	0.09	0.25	0.20	0.29	0.16	0.29
	0.57	0.68	0.60	0.40	0.89	0.11	0.82	0.40	0.83	0.61	0.89	0.65	0.90
K562_2	0.02	0.01	0.02	0.01	0.01	0.00	0.01	0.03	0.01	0.01	0.01	0.02	0.01
	0.91	0.75	0.90	0.78	0.75	0.25	0.67	0.82	0.51	0.48	0.75	0.72	0.71

Supplementary Table 16. Feature importance for the best performing full model. This table shows the AUPRC for the best performing full model (KNN trained in HepG2+K562_1) when each feature is removed in test datasets MCF7 and K562_2. The baseline AUPRC for MCF7 dataset=0.083. The baseline AUPRC for K562_2 dataset=0.005. The line highlighted in green shows the best performing final model, which contains all features except for Feature 11.

Feature removed	AUPRC (MCF7)	AUPRC (K562_2)
Feature 1: H3K27ac (Enhancer)	0.71	0.57
Feature 2: H3K4me1 (Enhancer)	0.66	0.45
Feature 3: H3K4me3 (Promoter)	0.71	0.50
Feature 4: P300 binding (Enhancer)	0.38	0.04
Feature 5: eQTL—combined Z-score	0.71	0.51
Feature 6: nearest gene	0.61	0.46
Feature 7: intronic	0.69	0.49
Feature 8: average of the absolute values of eQTL coefficients of eQTL located within the enhancer pointing to the same gene	0.70	0.50
Feature 9: H3K27ac (Promoter)	0.70	0.50
Feature 10: Feature1 x Feature2	0.71	0.46
Feature 11: Feature1 x Feature3	0.73	0.56
Feature 12: Feature1 x Feature9	0.71	0.56
Feature 13: Feature2 x Feature3	0.71	0.45
Feature 14: Feature2 x Feature9	0.70	0.50
Feature 15: Feature5 x Feature8	0.70	0.50
Feature 16: Feature1 x Feature2 x Feature3	0.72	0.46
Feature 17: Feature1 x Feature2 x Feature9	0.70	0.50

Supplementary Table 17. Feature importance for the final model. This table shows the AUPRC for the final model (KNN trained in HepG2+K562_1 without Feature 11) when each feature is removed in test datasets MCF7 and K562_2. The baseline AUPRC for MCF7 dataset=0.083. The baseline AUPRC for K562_2 dataset=0.005. Even though the AUPRC for the model without Feature 5 is marginally higher than the final model, the PR curves lost precision more rapidly with this model, so Feature 5 remained in the final model.

Feature removed	AUPRC (MCF7)	AUPRC (K562_2)
Feature 1: H3K27ac (Enhancer)	0.72	0.56
Feature 2: H3K4me1 (Enhancer)	0.67	0.56
Feature 3: H3K4me3 (Promoter)	0.74	0.49
Feature 4: P300 binding (Enhancer)	0.46	0.04
Feature 5: eQTL—combined Z-score	0.73	0.57
Feature 6: nearest gene	0.61	0.46
Feature 7: intronic	0.71	0.55
Feature 8: average of the absolute values of eQTL coefficients of eQTL located within the enhancer pointing to the same gene	0.72	0.50
Feature 9: H3K27ac (Promoter)	0.73	0.56
Feature 10: Feature1 x Feature2	0.72	0.56
Feature 11: Feature1 x Feature3		
Feature 12: Feature1 x Feature9	0.73	0.56
Feature 13: Feature2 x Feature3	0.74	0.56
Feature 14: Feature2 x Feature9	0.73	0.56
Feature 15: Feature5 x Feature8	0.73	0.56
Feature 16: Feature1 x Feature2 x Feature3	0.74	0.56
Feature 17: Feature1 x Feature2 x Feature9	0.73	0.56

Supplementary Figures.

Supplementary Figure 1. The genome-wide distribution of HepG2-specific PEACOCK scores generated using the final model. This figure shows the entire scoring distribution for all ~17M enhancer-gene pairs located within 1Mb of each other based on data generated from experiments in HepG2 cells. The scores have been log-transformed. For the sake of being on the histogram, scores of zero were changed to 0.0001.

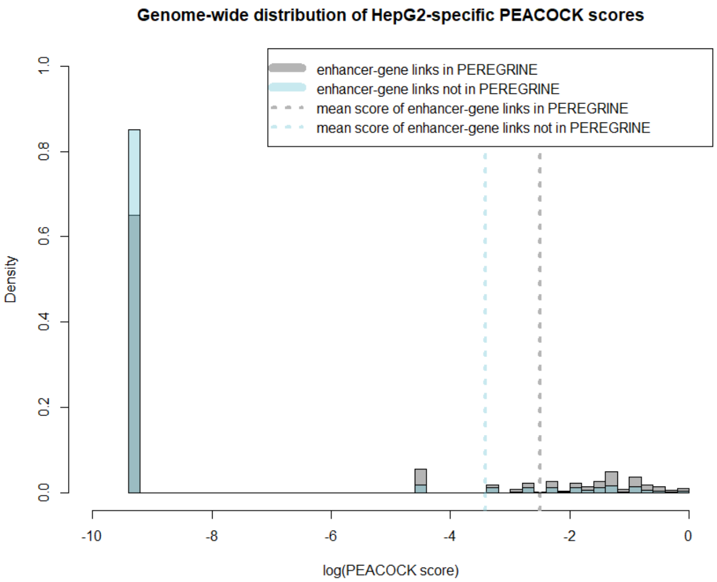

Supplementary Figure 2. The genome-wide distribution of HepG2-specific PEACOCK scores generated using the final model zoomed in to non-zero scores. This figure shows the scoring distribution for all ~17M enhancer-gene pairs located within 1Mb of each other, but zoomed in to show all scores>0. The scores have been log-transformed.

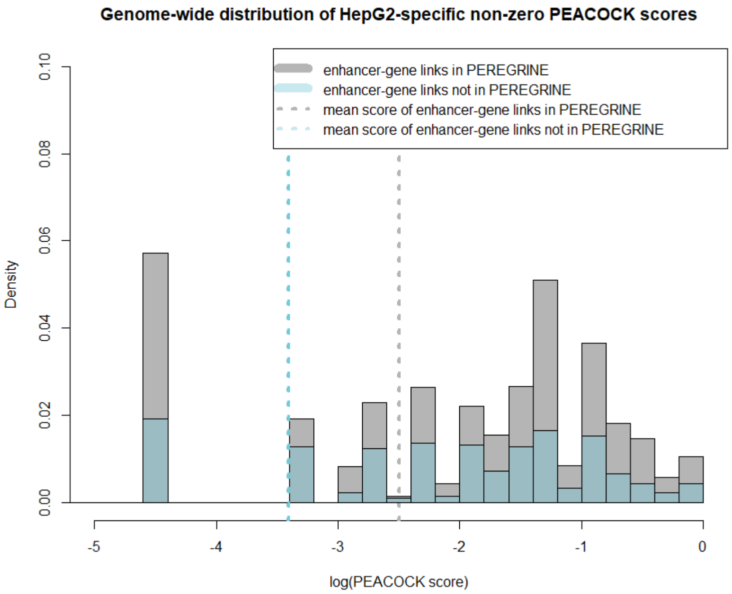

Supplementary Figure 3. The genome-wide distribution of HCT116-specific PEACOCK scores generated using the alternate final model. This figure shows the entire scoring distribution for all ~17M enhancer-gene pairs located within 1Mb of each other based on data generated from experiments in HCT116 cells. The scores have been log-transformed.

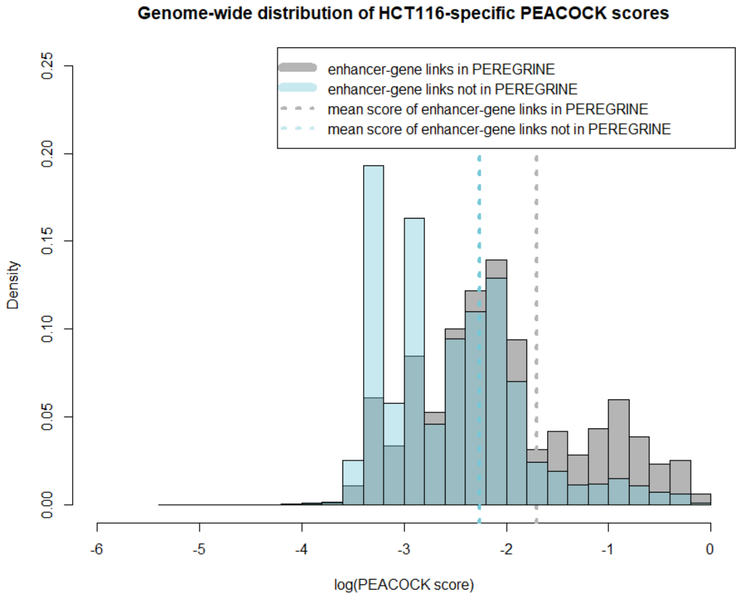

Supplementary Figure 4. The genome-wide distribution of K562-specific PEACOCK scores generated using the final model. This figure shows the entire scoring distribution for all ~17M enhancer-gene pairs located within 1Mb of each other based on data generated from experiments in K562 cells. The scores have been log-transformed. For the sake of being on the histogram, scores of zero were changed to 0.0001.

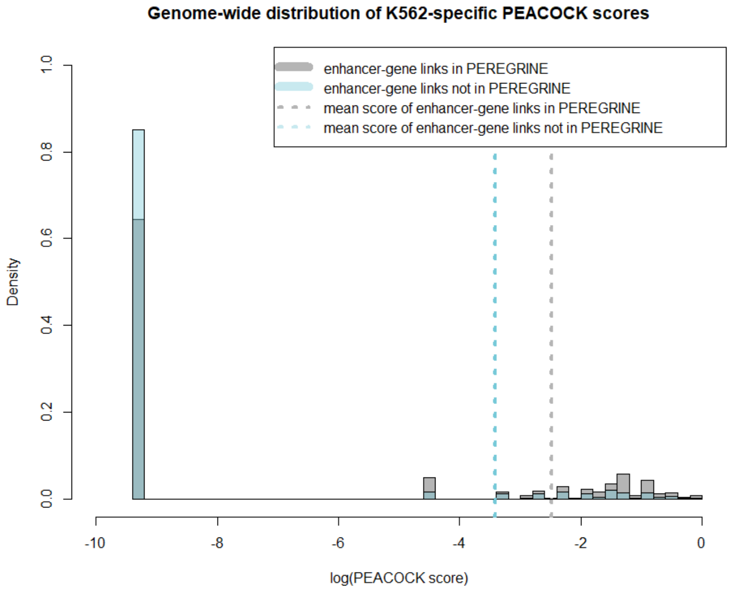

Supplementary Figure 5. The genome-wide distribution of K562-specific PEACOCK scores generated using the final model zoomed in to non-zero scores. This figure shows the scoring distribution for all ~17M enhancer-gene pairs located within 1Mb of each other, but zoomed in to show all scores>0. The scores have been log-transformed.

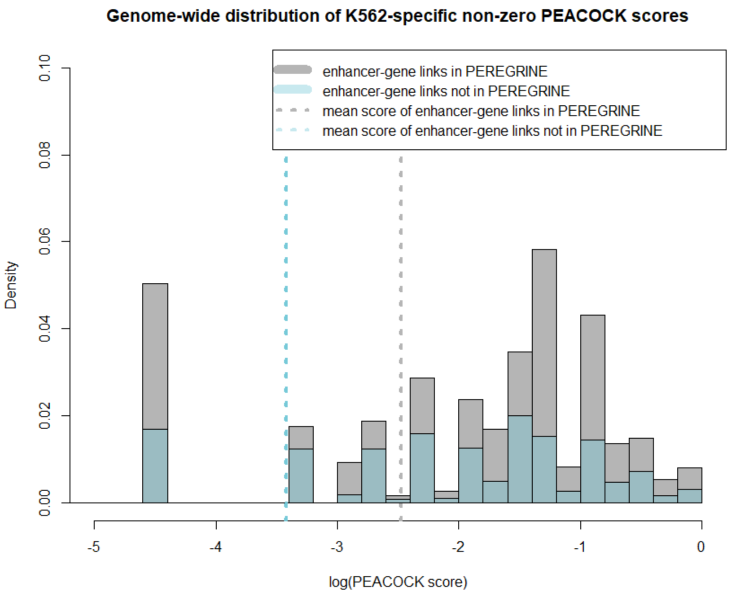

Supplementary Figure 6. The genome-wide distribution of MCF7-specific PEACOCK scores generated using the final model. This figure shows the entire scoring distribution for all ~17M enhancer-gene pairs located within 1Mb of each other based on data generated from experiments in MCF7 cells. The scores have been log-transformed. For the sake of being on the histogram, scores of zero were changed to 0.0001.

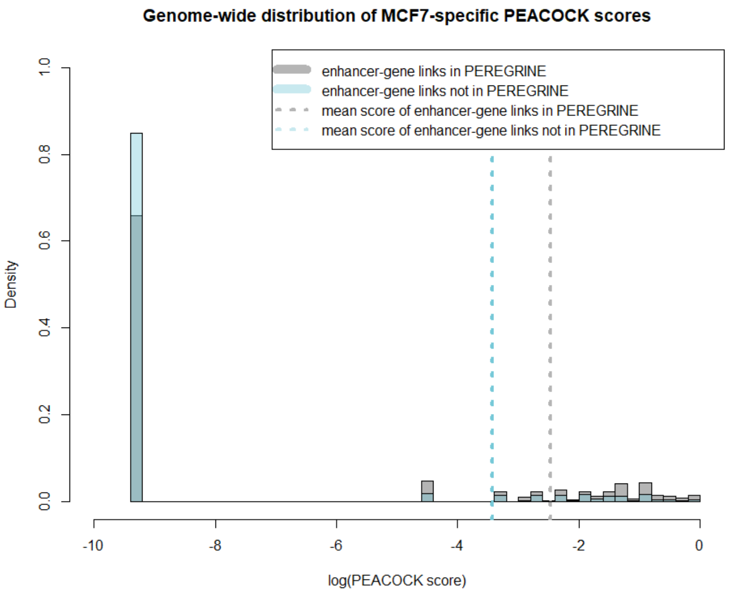

Supplementary Figure 7. The genome-wide distribution of MCF7-specific PEACOCK scores generated using the final model zoomed in to non-zero scores. This figure shows the scoring distribution for all ~17M enhancer-gene pairs located within 1Mb of each other, but zoomed in to show all scores>0. The scores have been log-transformed.

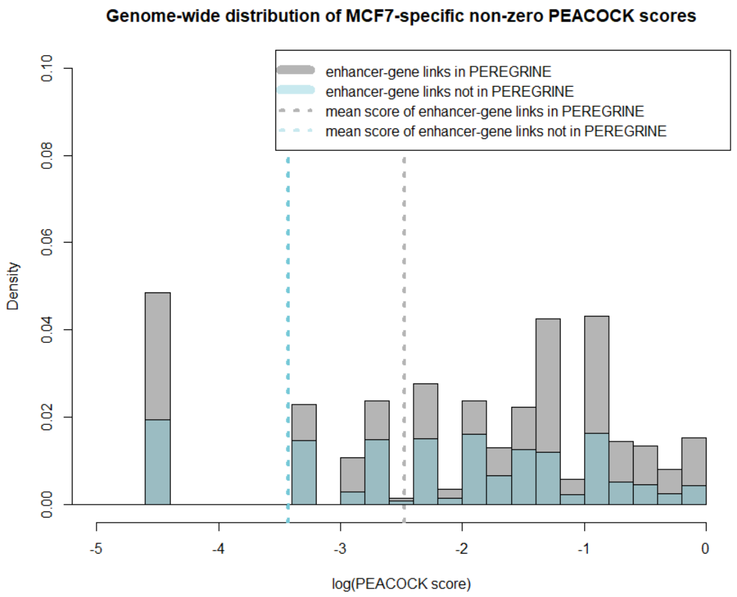

Supplementary Figure 8. Precision-recall curves of PEACOCK and ABC. This figure plots the PRC of all observations in K562_2 data with predictions available in both PEACOCK and ABC. AUPRC=0.68 for PEACOCK prediction scores, and AUPRC=0.47 for ABC prediction scores.

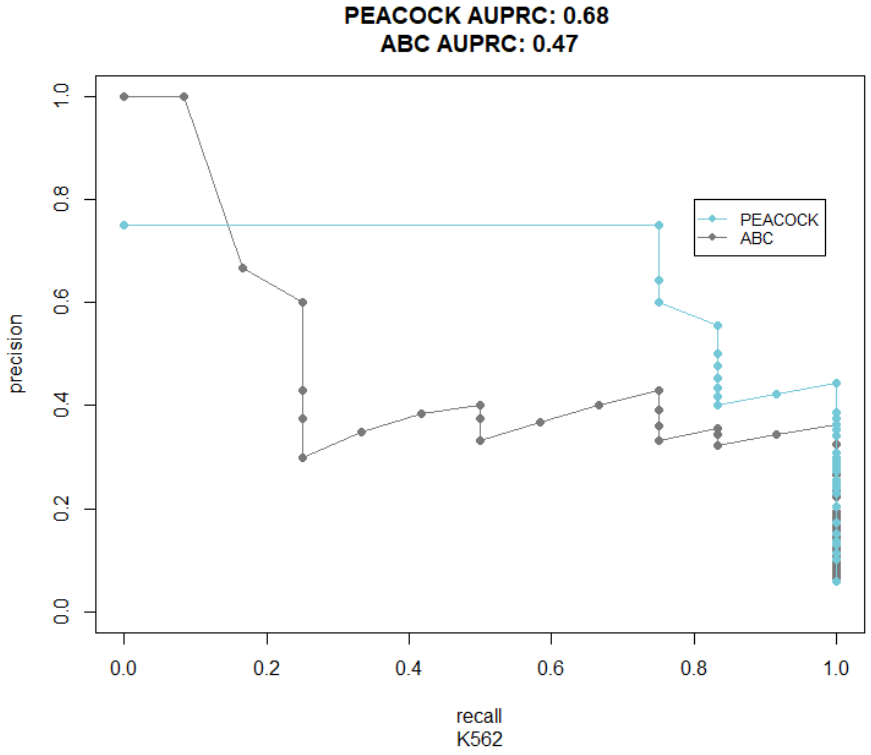

Supplementary Figure 9. Precision-recall curves of PEACOCK and GeneHancer. This figure plots the PRC of all observations in HCT116, K562_2, and MCF7 data with predictions available in both PEACOCK and GeneHancer. AUPRC=0.45 for PEACOCK prediction scores, and AUPRC=0.04 for GeneHancer prediction scores.

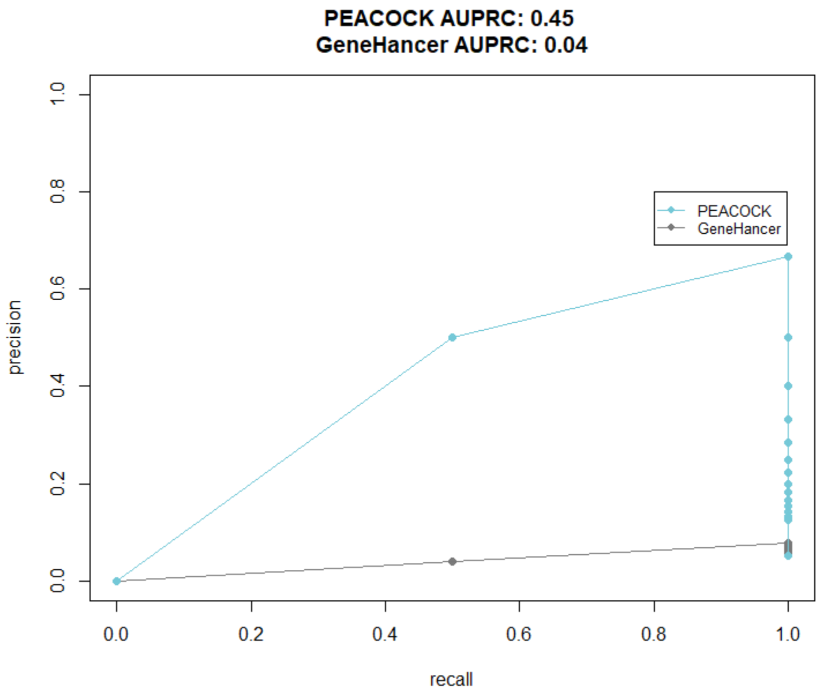

Supplementary Figure 10. Precision-recall curves of PEACOCK and TargetFinder. This figure plots the PRC of all observations in K562_2 data with predictions available in both PEACOCK and TargetFinder. AUPRC=0.33 for PEACOCK prediction scores, and AUPRC=0.04 for TargetFinder prediction scores.

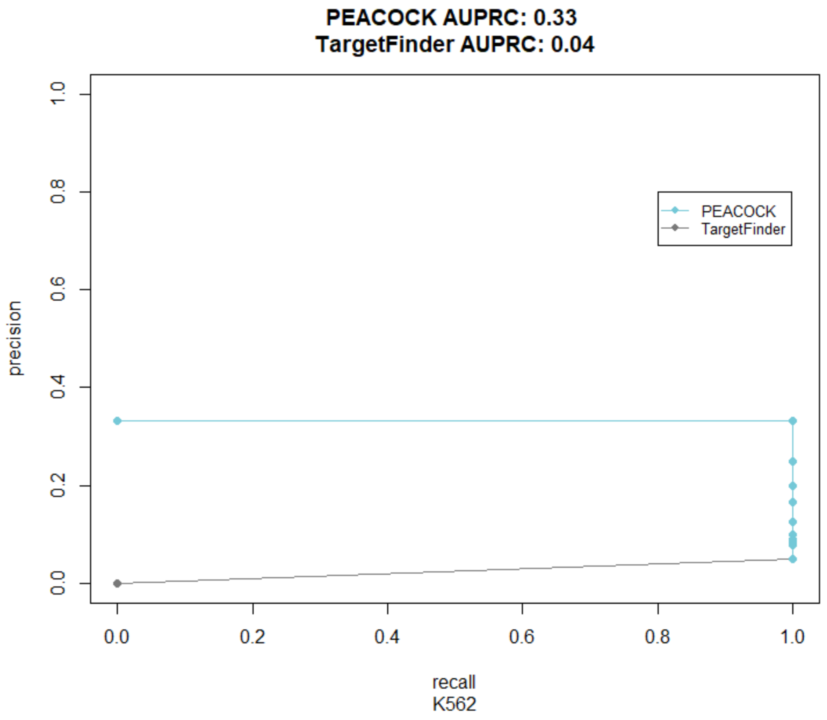

Supplementary Figure 11. Scores in K562 versus HepG2. This figure takes 50,000 randomly sample enhancer-gene pairs from among the 17M scored in PEACOCK and plots the K562-specific score against the HepG2-specific score for each pair.

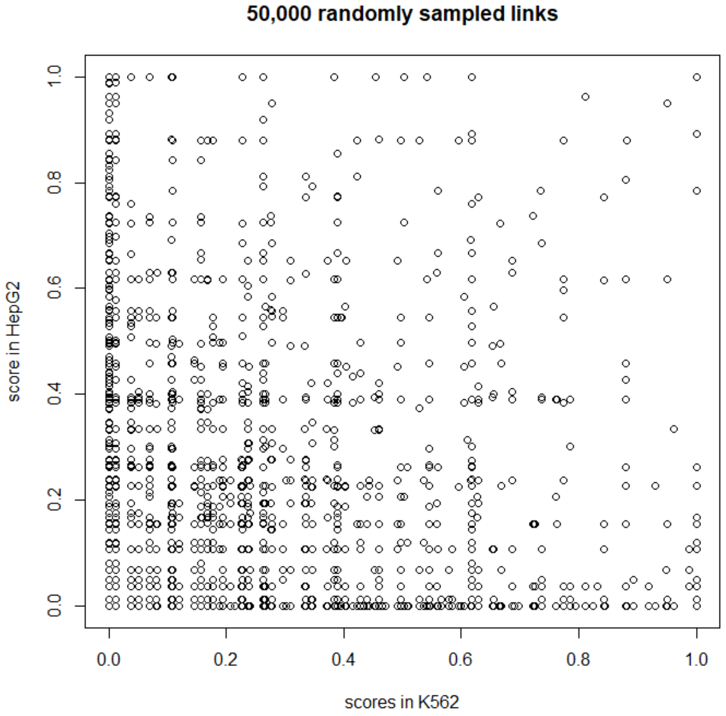

Supplementary Figure 12. Scores in K562 versus HCT116. This figure takes 50,000 randomly sample enhancer-gene pairs from among the 17M scored in PEACOCK and plots the K562-specific score against the HCT116-specific score for each pair.

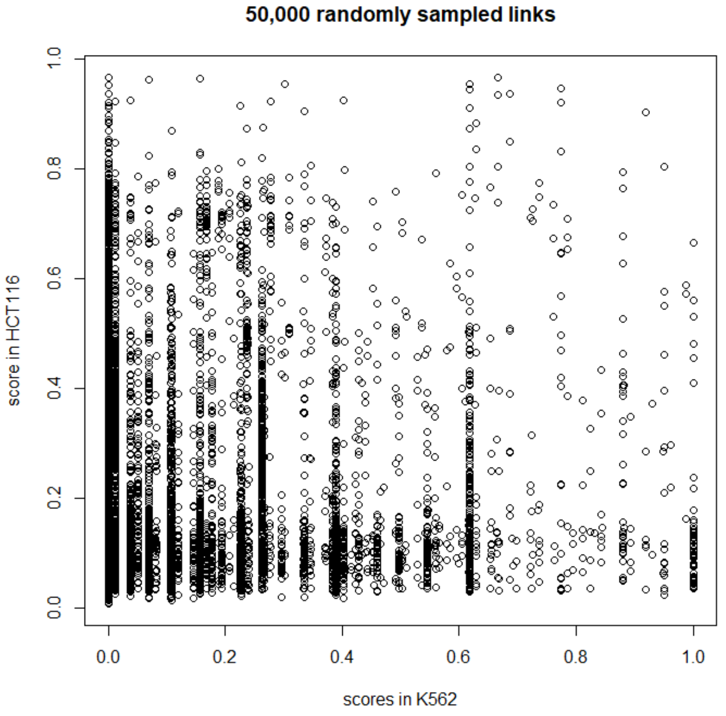

Supplementary Figure 13. Scores in K562 versus MCF7. This figure takes 50,000 randomly sample enhancer-gene pairs from among the 17M scored in PEACOCK and plots the K562-specific score against the MCF7-specific score for each pair.

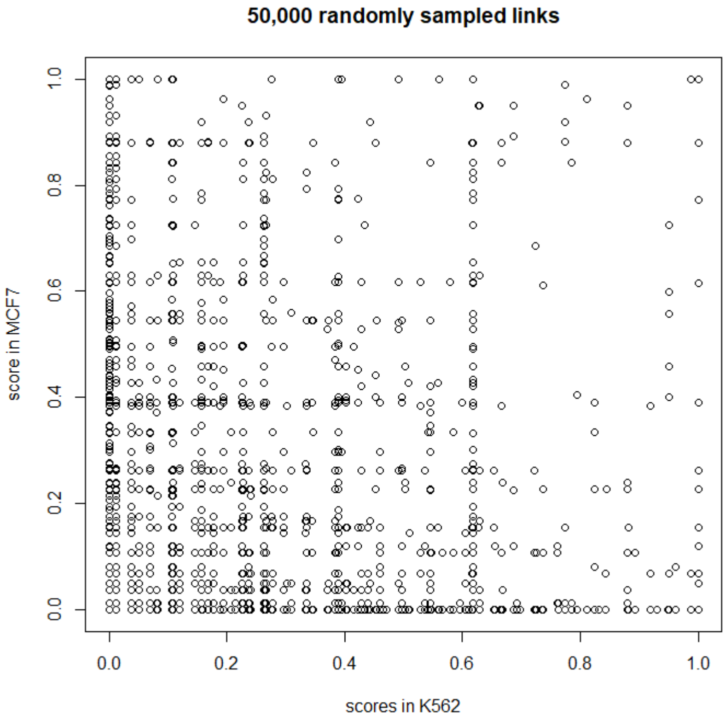

Supplementary Figure 14. Scores in HCT116 versus HepG2. This figure takes 50,000 randomly sample enhancer-gene pairs from among the 17M scored in PEACOCK and plots the HCT116-specific score against the HepG2-specific score for each pair.

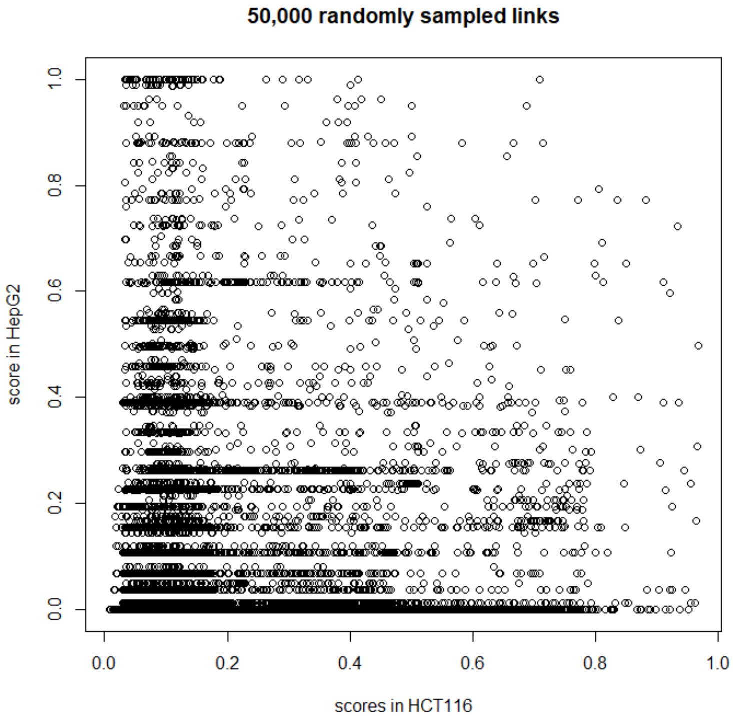

Supplementary Figure 15. Scores in HCT116 versus MCF7. This figure takes 50,000 randomly sample enhancer-gene pairs from among the 17M scored in PEACOCK and plots the HCT116-specific score against the MCF7-specific score for each pair.

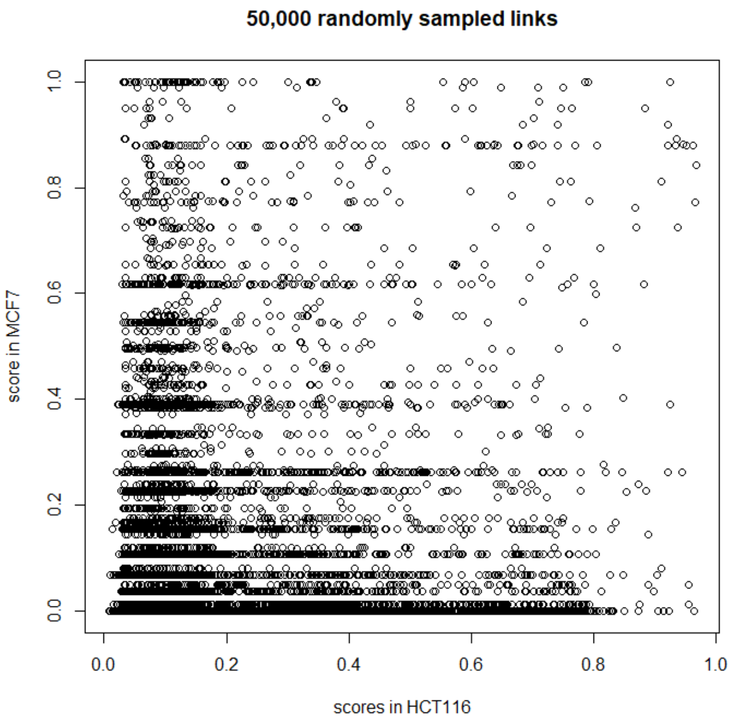

Supplementary Figure 16. Scores in MCF7 versus HepG2. This figure takes 50,000 randomly sample enhancer-gene pairs from among the 17M scored in PEACOCK and plots the MCF7-specific score against the HepG2-specific score for each pair.

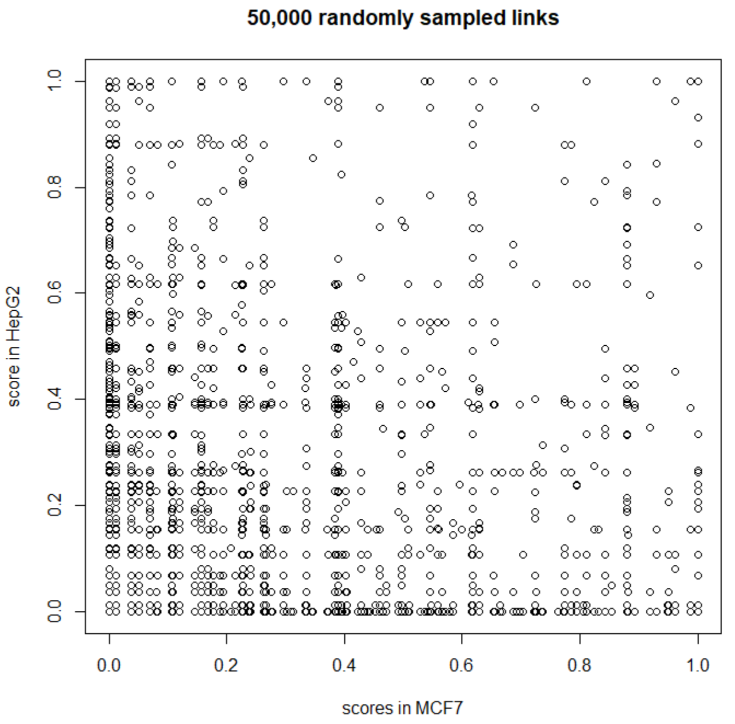

Supplementary Figure 17. Cell-specific scoring distributions for genome-wide non-zero scores. The histogram showing the scoring distribution for each of the three cell lines scored with the final model (HepG2, HCT116, and K562) are overlaid for comparison. The log-transformed scores are shown. HCT116 was scored using the alternate final model and is therefore excluded from this plot.

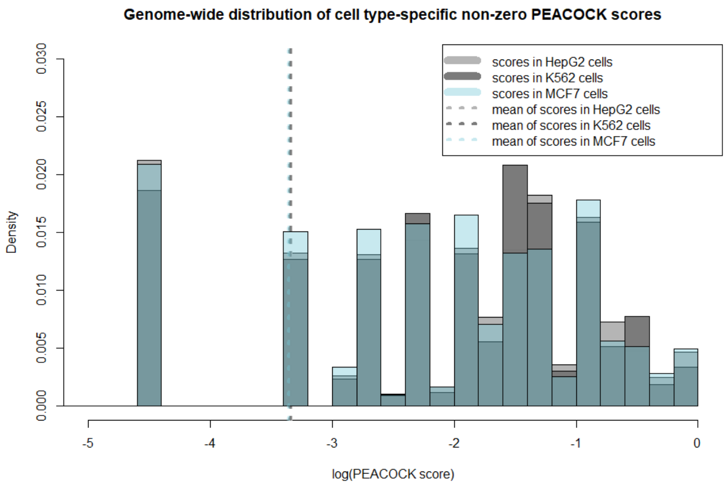

Supplementary Figure 18. Precision-recall curves for models trained in HepG2. This figure shows the precision-recall curves for every available testing set overlaid for models M1-M13 trained on the HepG2 dataset with the omission of the SVM with Bessel kernel, for convenience of displaying all plots in the same window and due to the fact that it was almost always the lowest performing model.

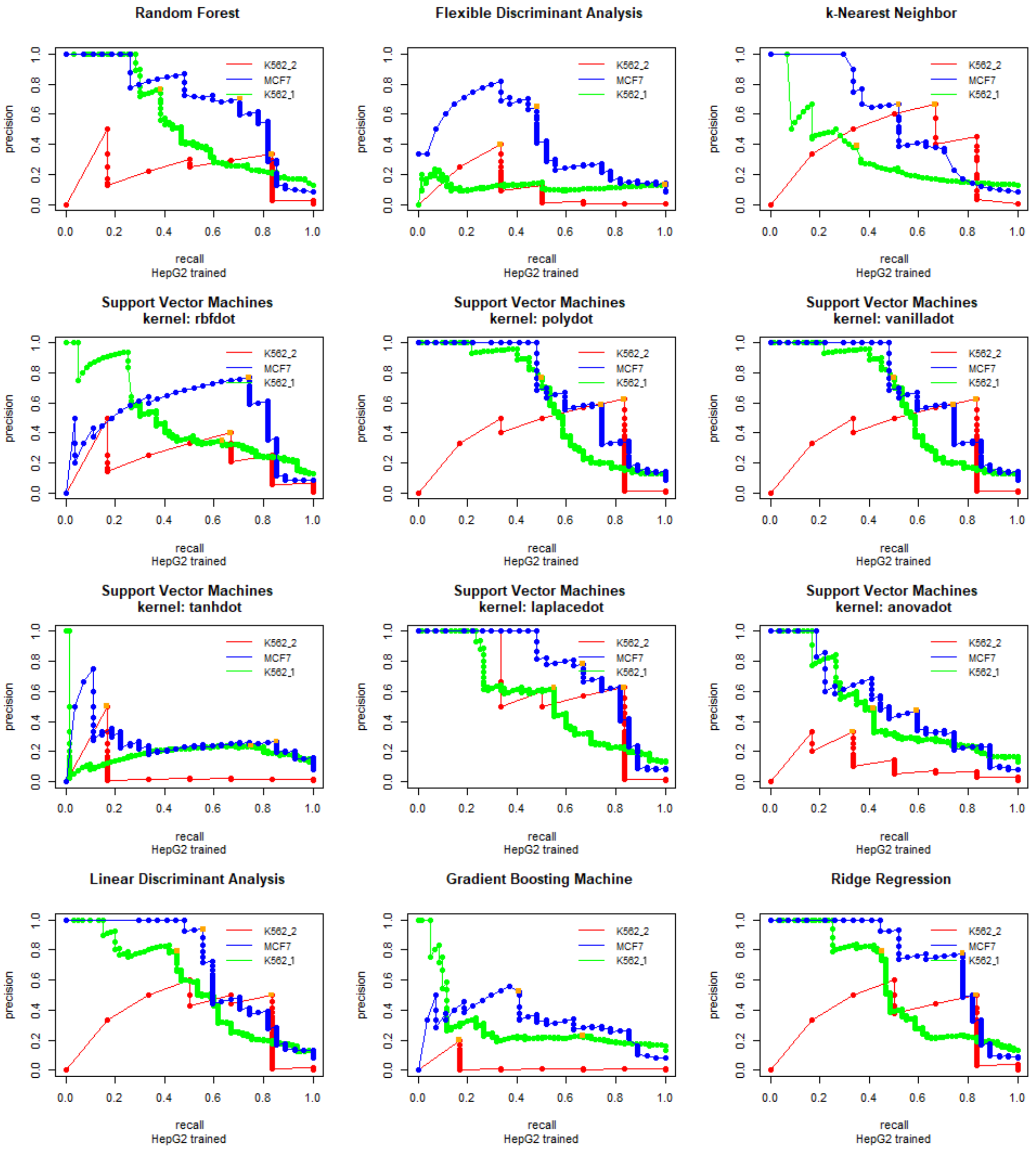

Supplementary Figure 19. Receiver operating characteristic curves for models trained in HepG2. This figure shows the ROC curves for every available testing set overlaid for models M1-M13 trained on the HepG2 dataset with the omission of the SVM with Bessel kernel, for convenience of displaying all plots in the same window and due to the fact that it was almost always the lowest performing model.

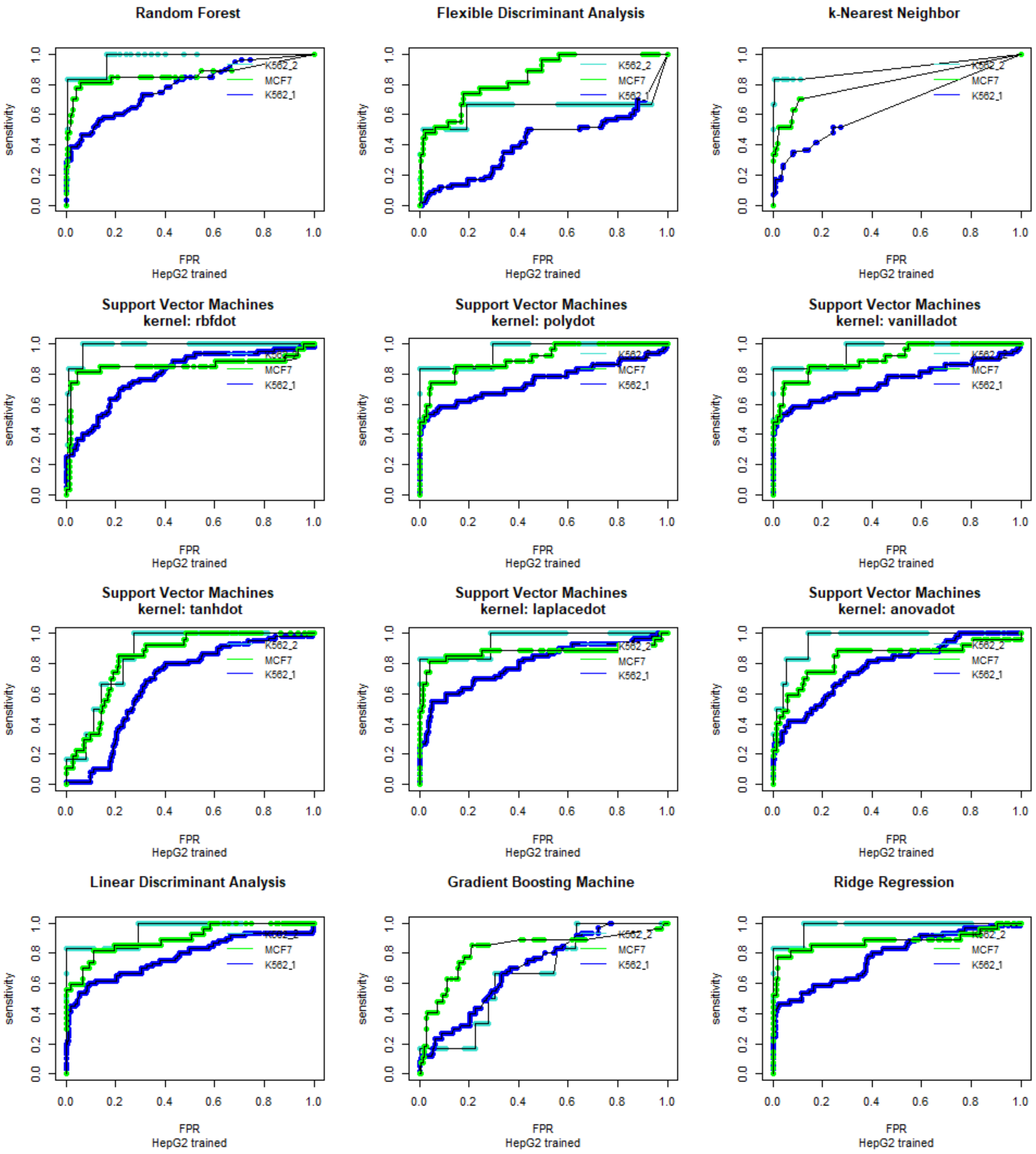

Supplementary Figure 20. Precision-recall curves for models trained in HCT116. This figure shows the precision-recall curves for every available testing set overlaid for models M1-M13 trained on the HCT116 dataset with the omission of the SVM with Bessel kernel, for convenience of displaying all plots in the same window and due to the fact that it was almost always the lowest performing model.

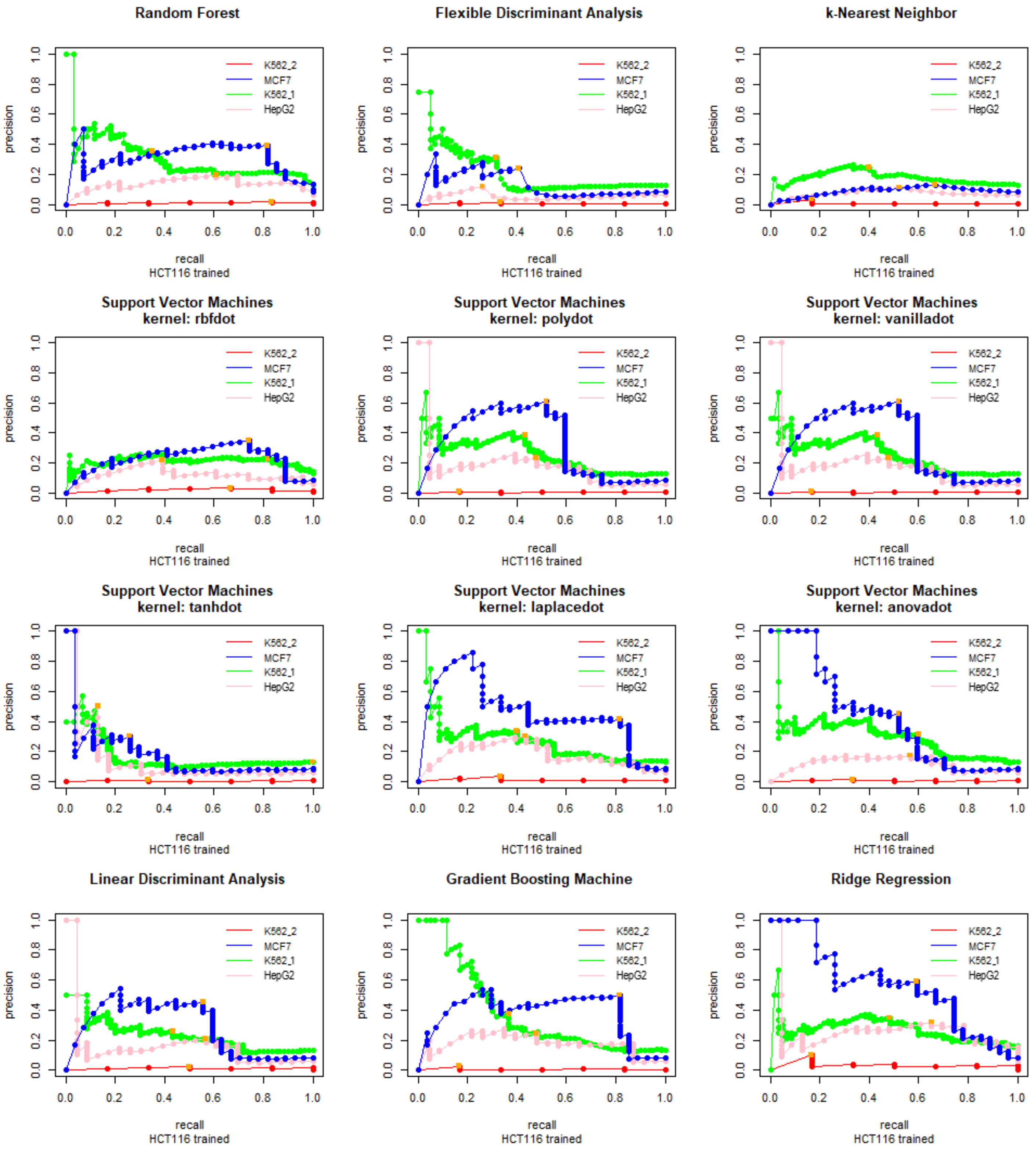

Supplementary Figure 21. Receiver operating characteristic curves for models trained in HCT116. This figure shows the ROC curves for every available testing set overlaid for models M1-M13 trained on the HCT116 dataset with the omission of the SVM with Bessel kernel, for convenience of displaying all plots in the same window and due to the fact that it was almost always the lowest performing model.

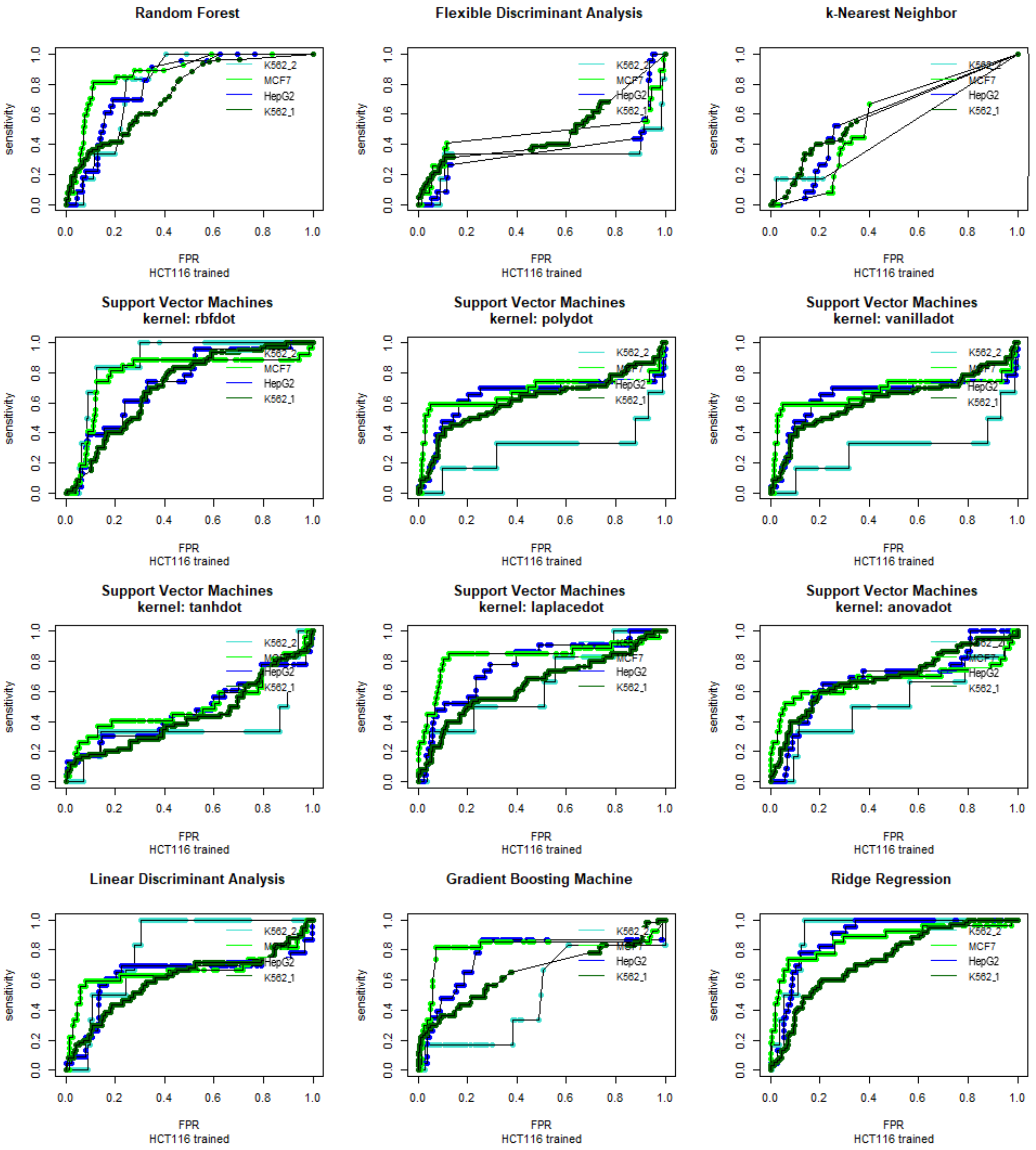

Supplementary Figure 22. Precision-recall curves for models trained in K562_1. This figure shows the precision-recall curves for every available testing set overlaid for models M1-M13 trained on the K562_1 dataset with the omission of the SVM with Bessel kernel, for convenience of displaying all plots in the same window and due to the fact that it was almost always the lowest performing model.

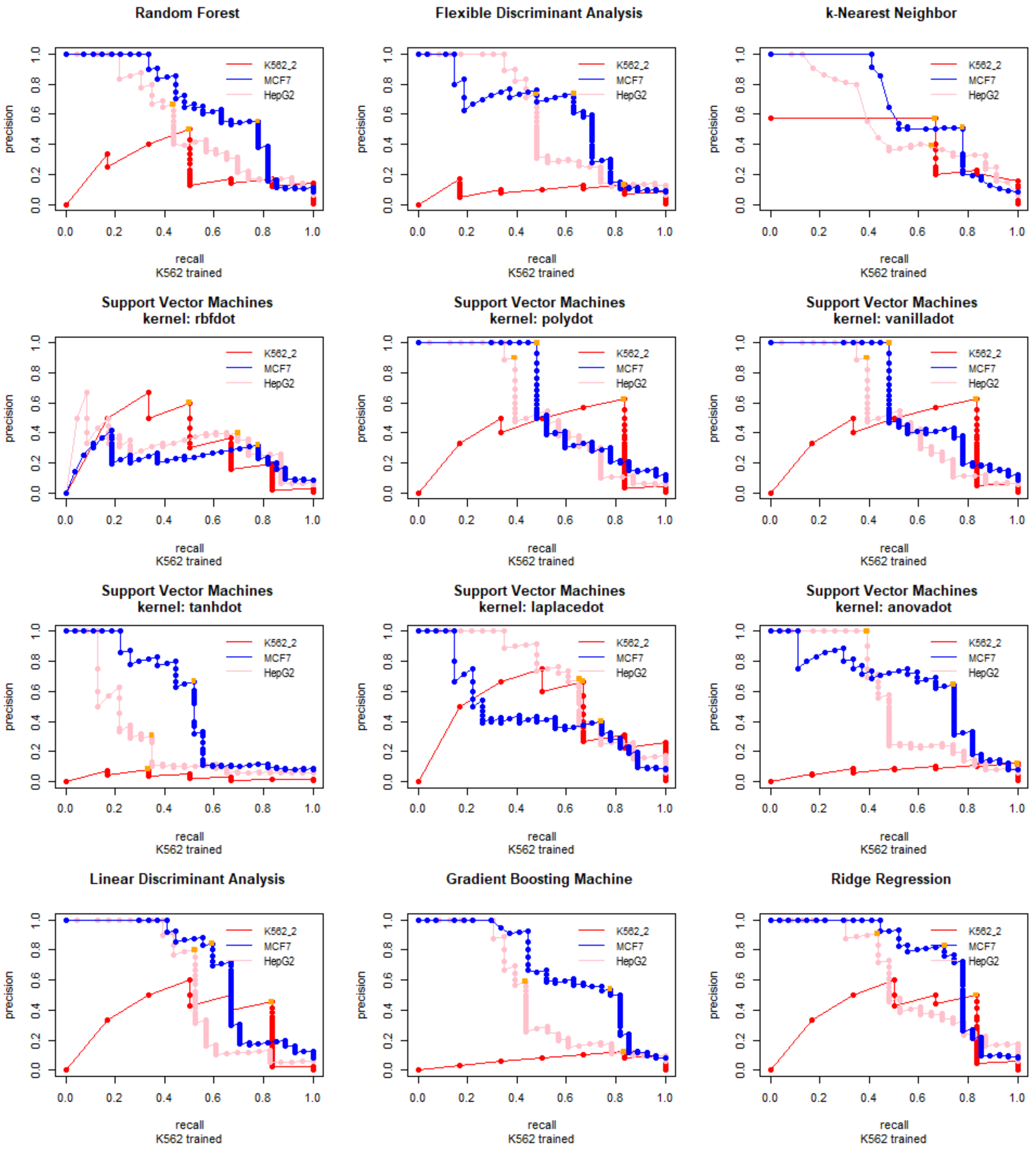

Supplementary Figure 23. Receiver operating characteristic curves for models trained in K562_1. This figure shows the ROC curves for every available testing set overlaid for models M1-M13 trained on the K562_1 dataset with the omission of the SVM with Bessel kernel, for convenience of displaying all plots in the same window and due to the fact that it was almost always the lowest performing model.

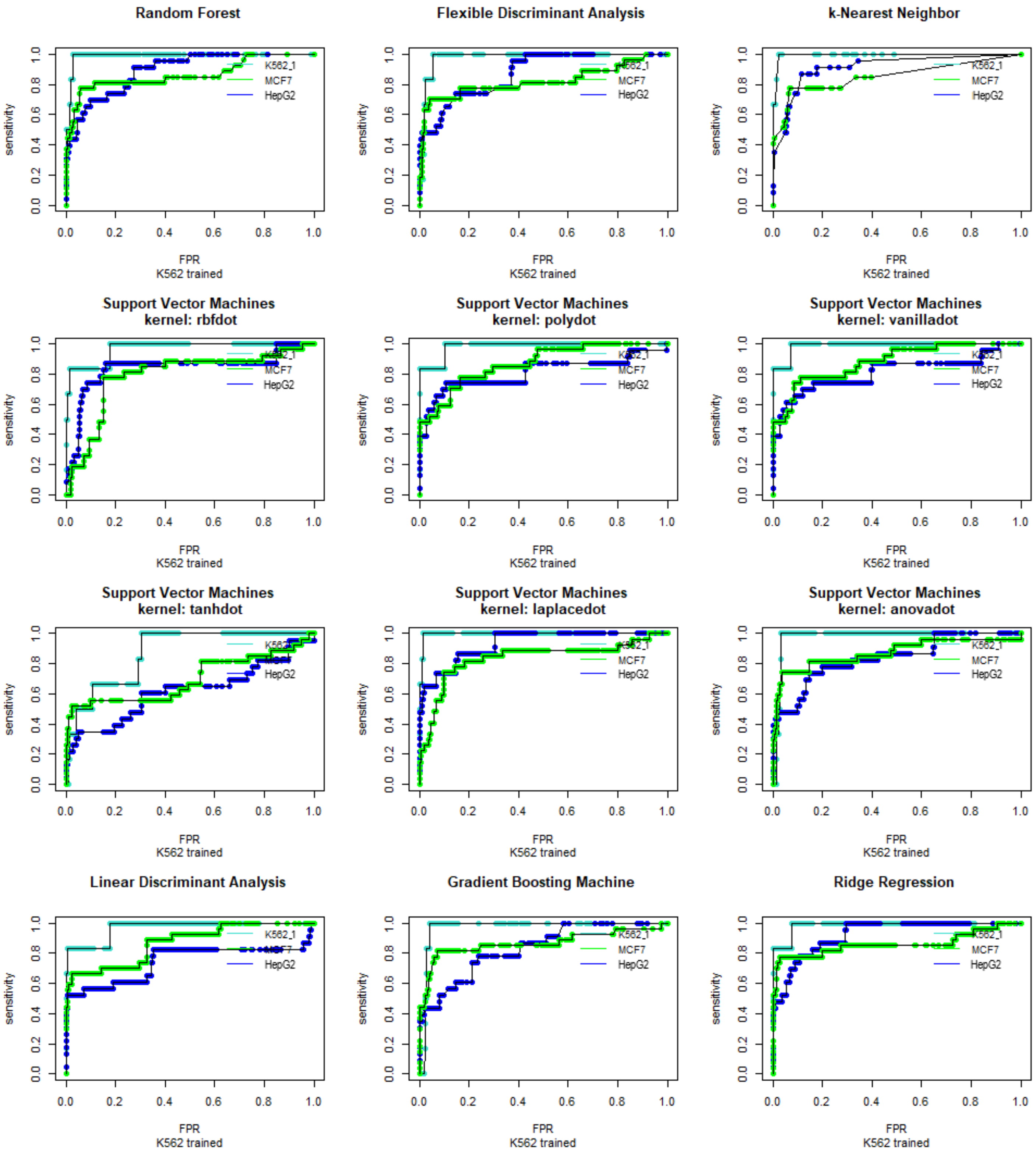

Supplementary Figure 24. Precision-recall curves for models trained in HepG2+HCT116. This figure shows the precision-recall curves for every available testing set overlaid for models M1-M13 trained on the HepG2+HCT116 joint dataset with the omission of the SVM with Bessel kernel, for convenience of displaying all plots in the same window and due to the fact that it was almost always the lowest performing model.

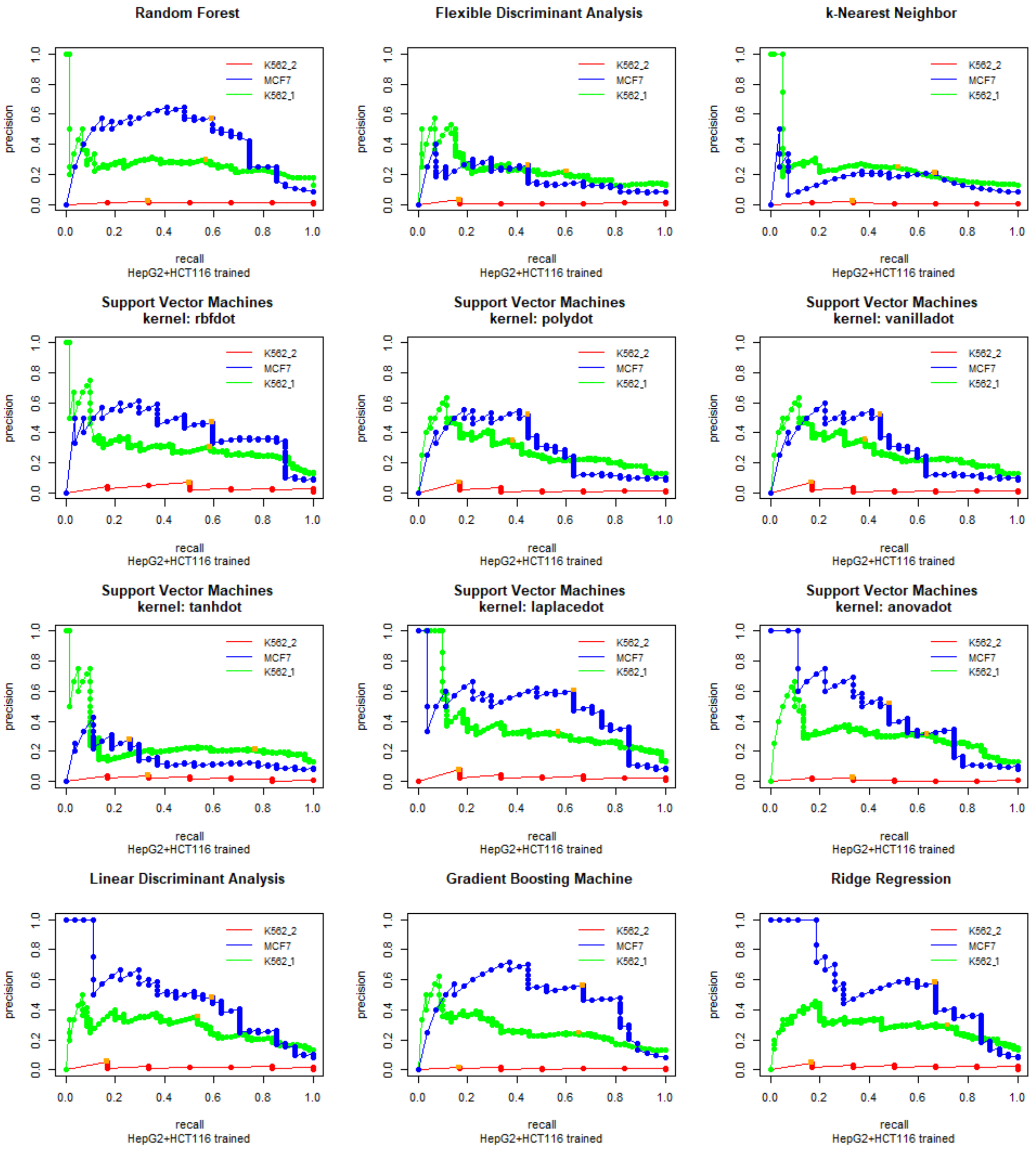

Supplementary Figure 25. Receiver operating characteristic curves for models trained in HepG2+HCT116. This figure shows the ROC curves for every available testing set overlaid for models M1-M13 trained on the HepG2+HCT116 dataset with the omission of the SVM with Bessel kernel, for convenience of displaying all plots in the same window and due to the fact that it was almost always the lowest performing model.

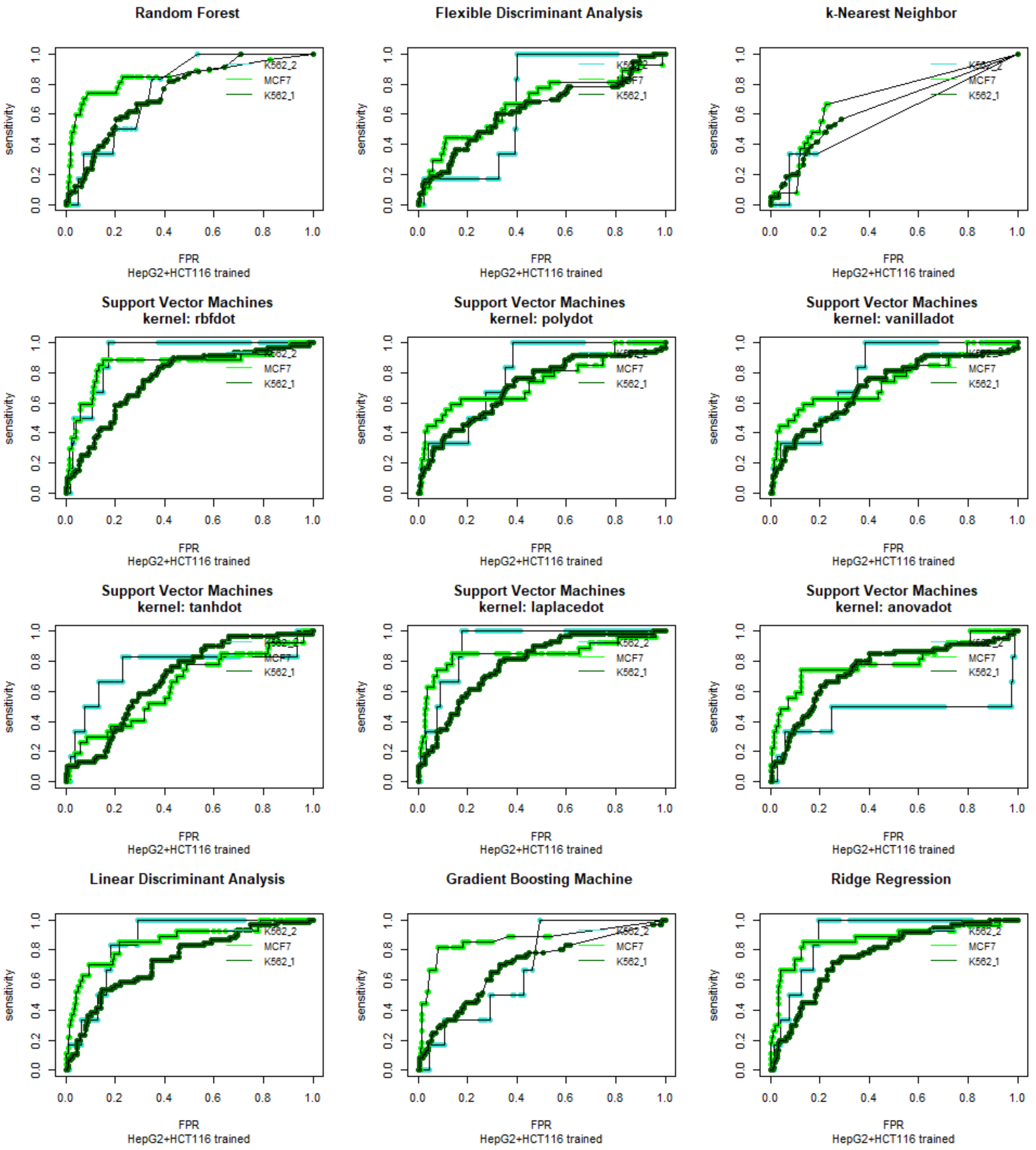

Supplementary Figure 26. Precision-recall curves for models trained in HepG2+K562_1. This figure shows the precision-recall curves for every available testing set overlaid for models M1-M13 trained on the HepG2+K562_1 joint dataset with the omission of the SVM with Bessel kernel, for convenience of displaying all plots in the same window and due to the fact that it was almost always the lowest performing model.

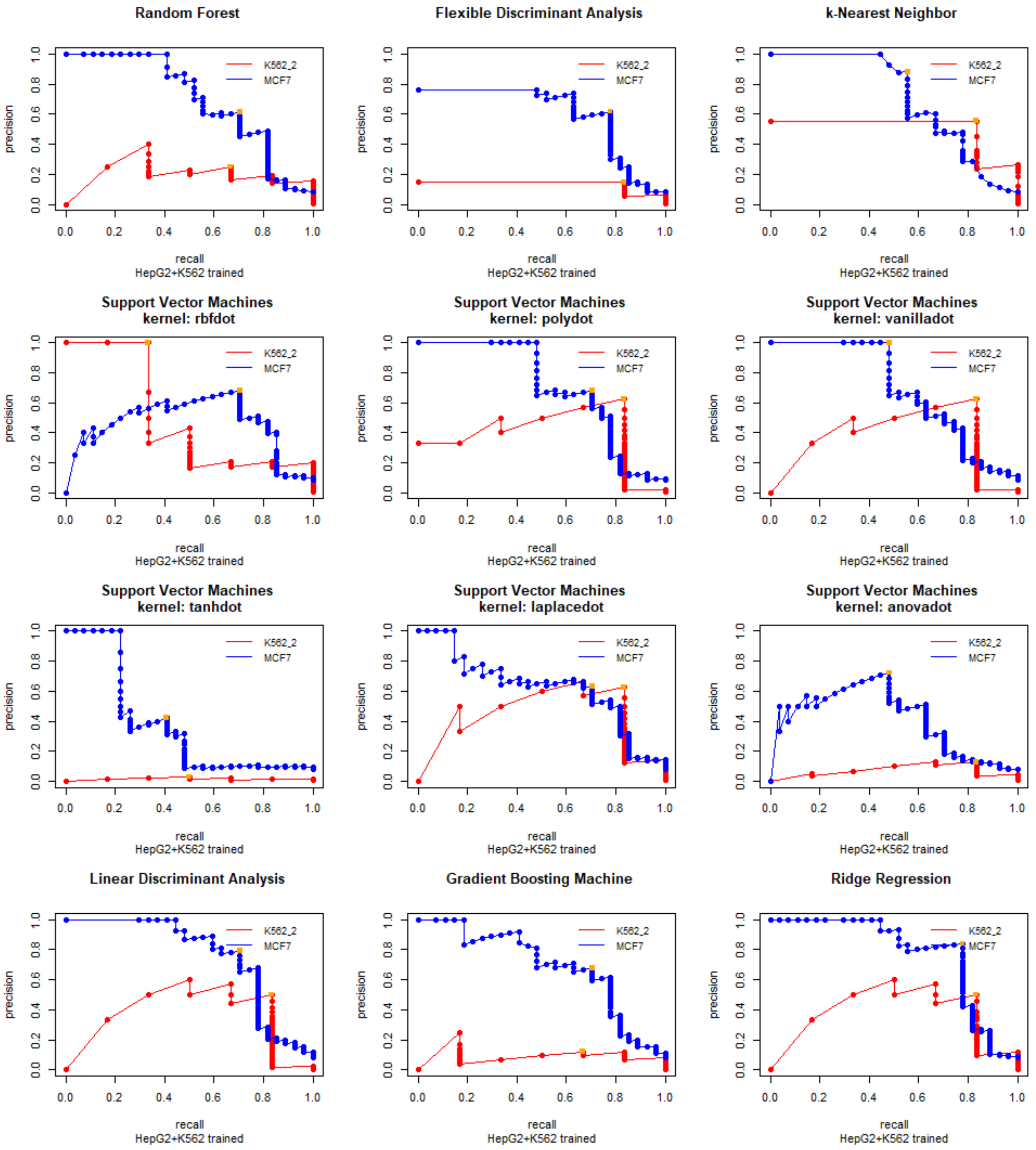

Supplementary Figure 27. Receiver operating characteristic curves for models trained in HepG2+K562_1. This figure shows the ROC curves for every available testing set overlaid for models M1-M13 trained on the HepG2+K562_1 joint dataset with the omission of the SVM with Bessel kernel, for convenience of displaying all plots in the same window and due to the fact that it was almost always the lowest performing model.

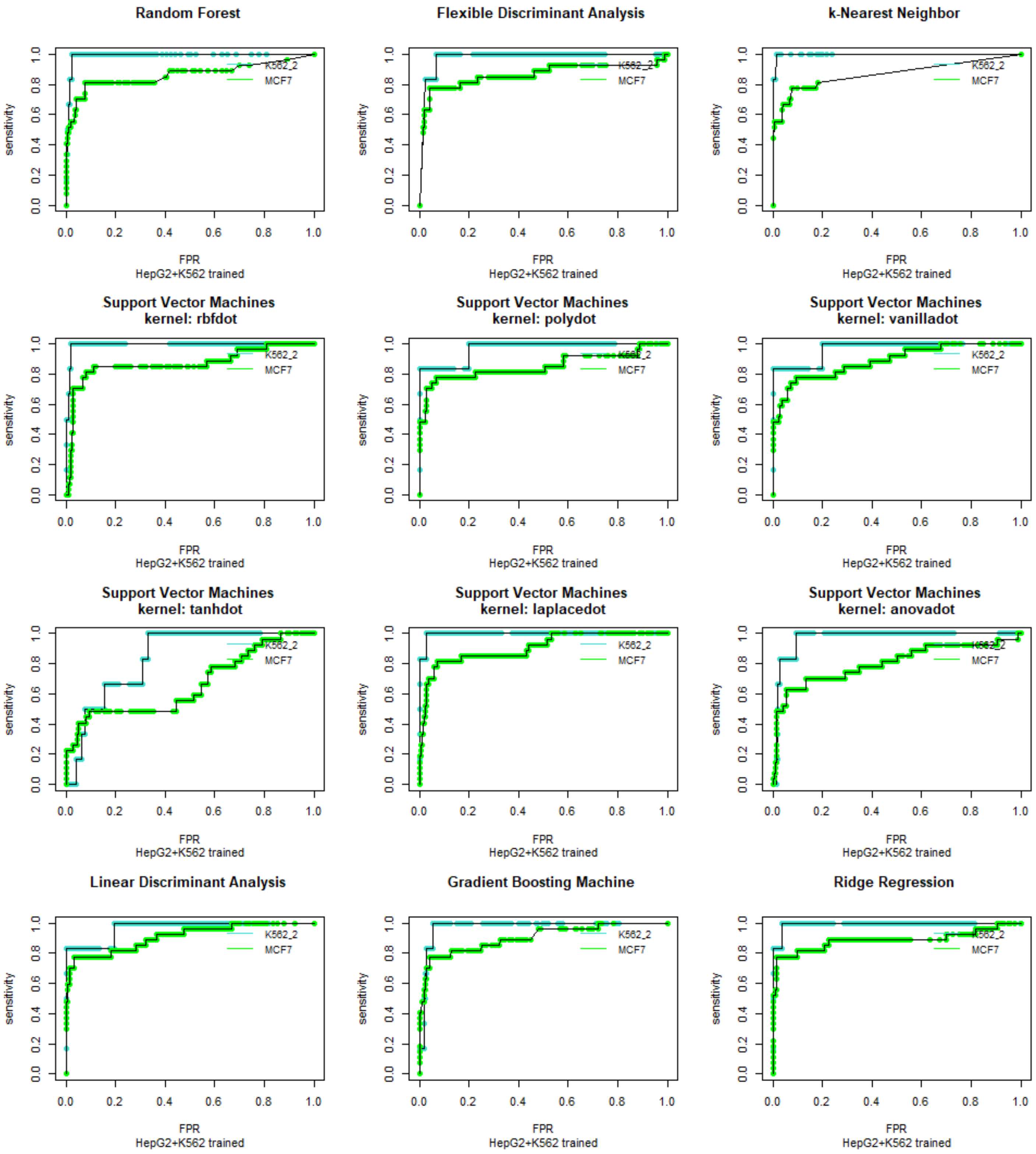

Supplementary Figure 28. Precision-recall curves for models trained in HCT116+K562_1. This figure shows the precision-recall curves for every available testing set overlaid for models M1-M13 trained on the HCT116+K562_1 joint dataset with the omission of the SVM with Bessel kernel, for convenience of displaying all plots in the same window and due to the fact that it was almost always the lowest performing model.

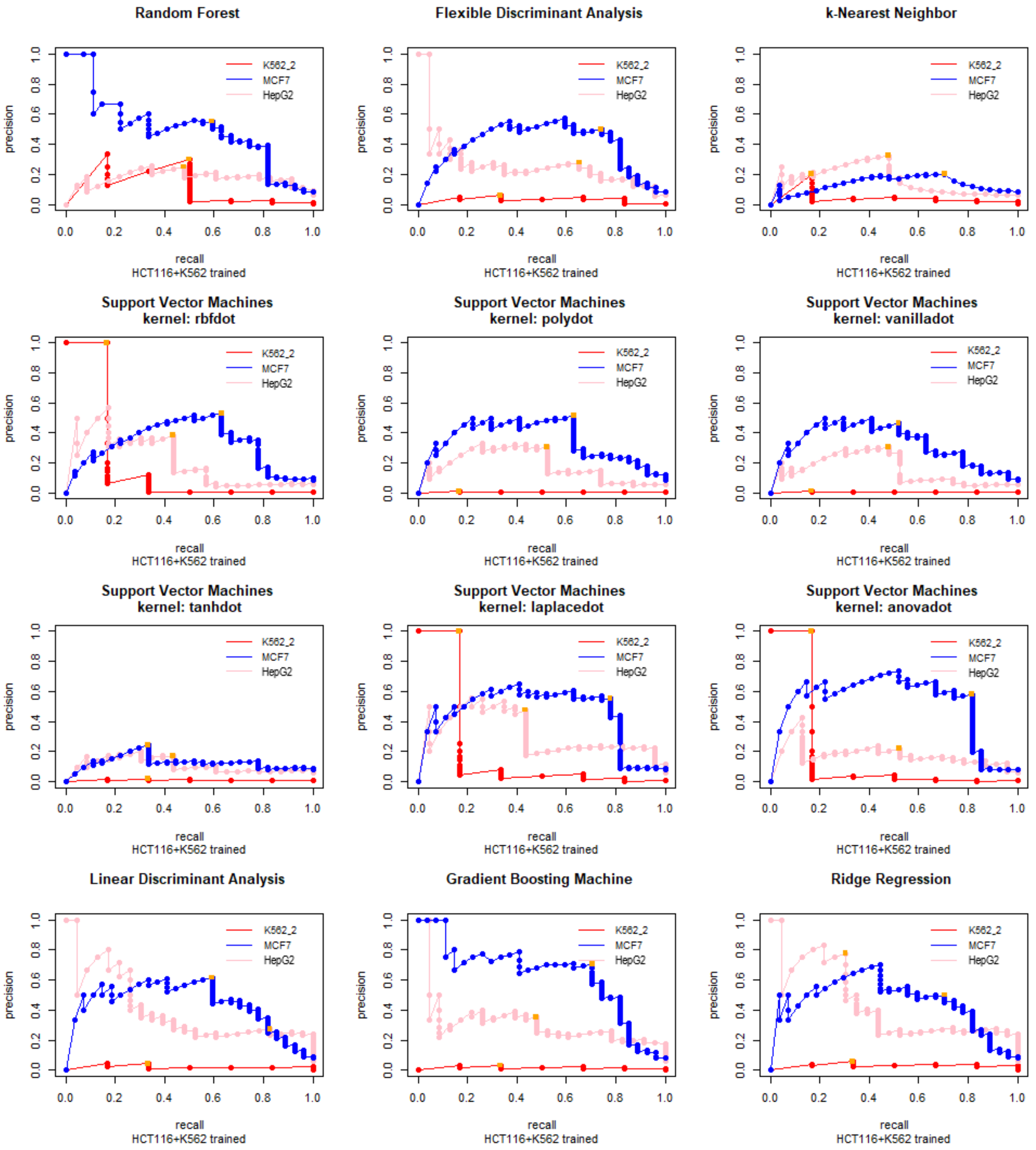

Supplementary Figure 29. Receiver operating characteristic curves for models trained in HCT116+K562_1. This figure shows the ROC curves for every available testing set overlaid for models M1-M13 trained on the HCT116+K562_1 joint dataset with the omission of the SVM with Bessel kernel, for convenience of displaying all plots in the same window and due to the fact that it was almost always the lowest performing model.

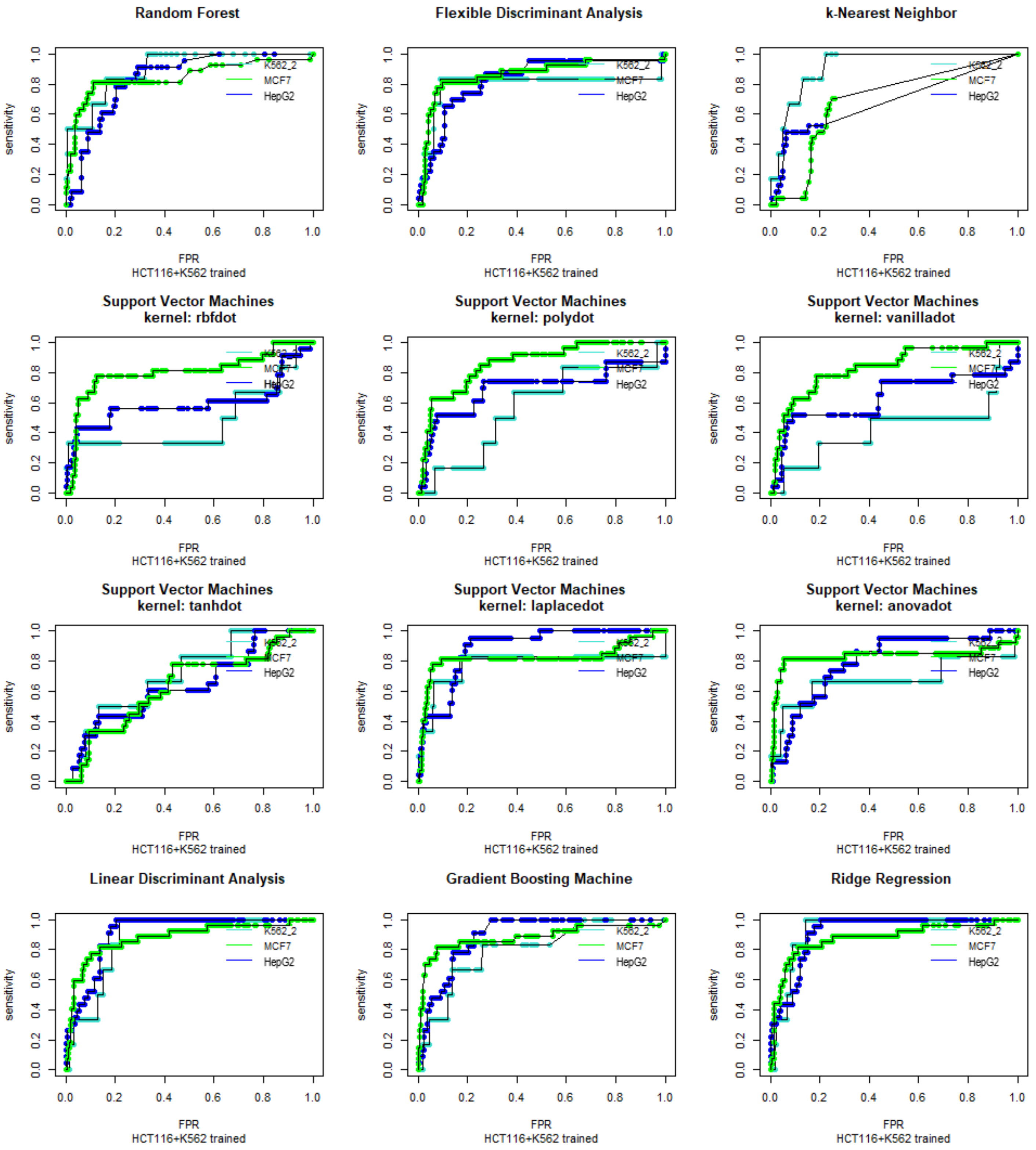

Supplementary Figure 30. Precision-recall curves for models trained in HepG2+HCT116+K562_1. This figure shows the precision-recall curves for every available testing set overlaid for models M1-M13 trained on the HepG2+HCT116+K562_1 joint dataset with the omission of the SVM with Bessel kernel, for convenience of displaying all plots in the same window and due to the fact that it was almost always the lowest performing model.

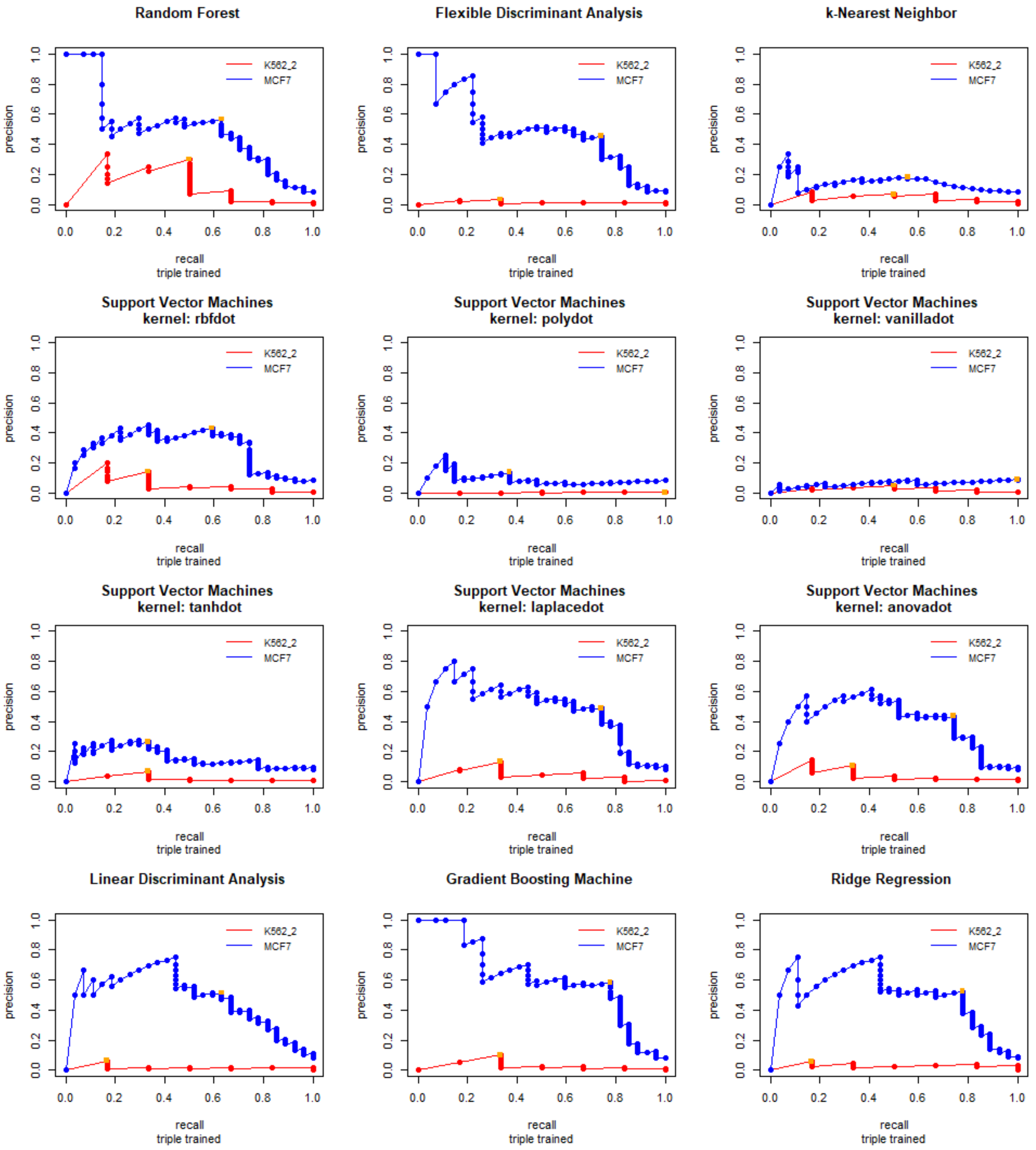

Supplementary Figure 31. Receiver operating characteristic curves for models trained in HepG2+HCT116+K562_1. This figure shows the ROC curves for every available testing set overlaid for models M1-M13 trained on the HepG2 dataset with the omission of the SVM with Bessel kernel, for convenience of displaying all plots in the same window and due to the fact that it was almost always the lowest performing model.

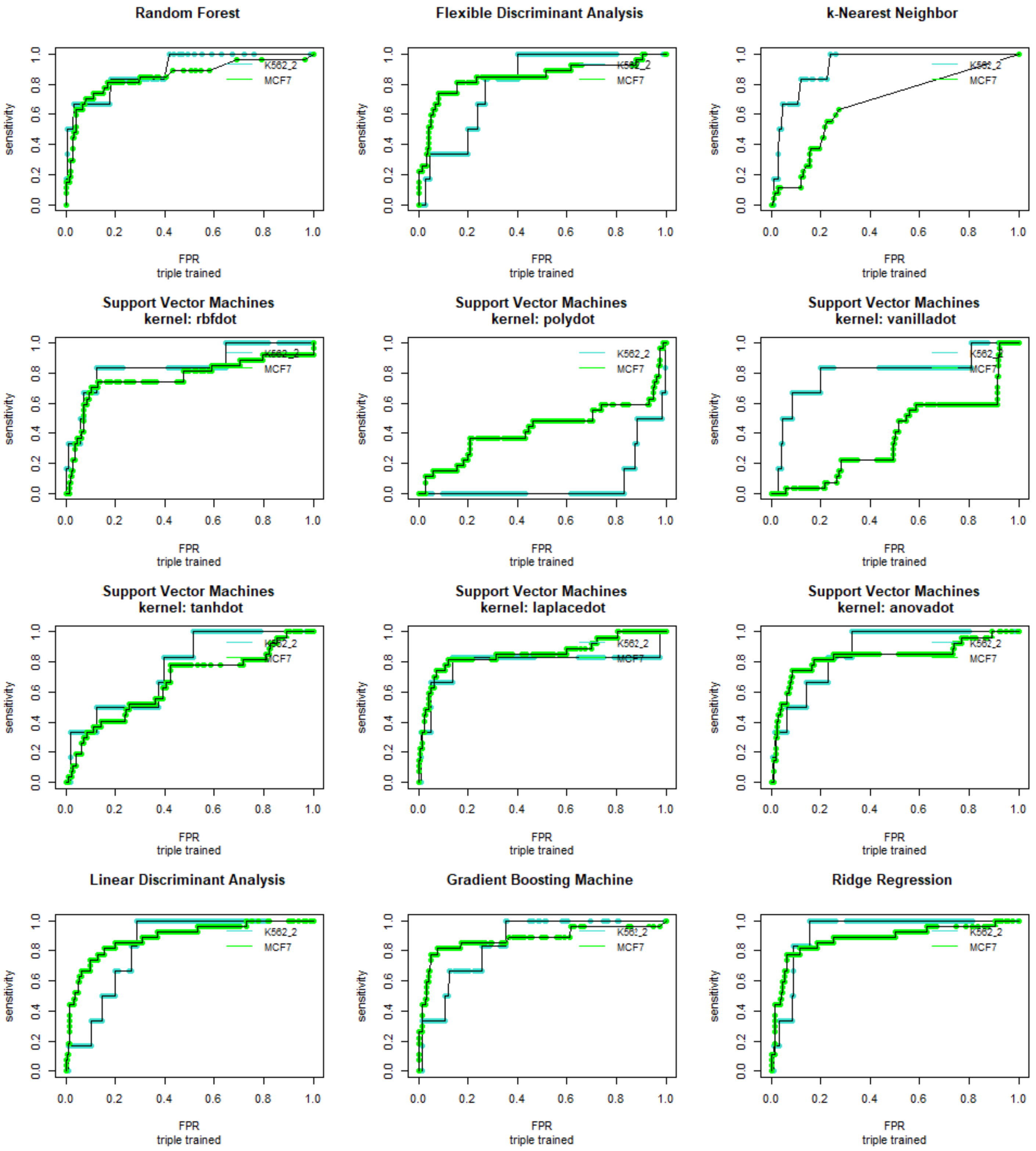

Supplement: Supplementary file 1 — Supplemental Materials [file 41540_2023_270_MOESM1_ESM.pdf]
